# Assessing the proinflammatory potential of sterile fecal microbiome filtrate from ulcerative colitis patients using an intestine-on-chip platform and automated image analysis

**DOI:** 10.1080/19490976.2026.2701400

**Published:** 2026-07-28

**Authors:** Tobias Schaal, Parastoo Akbarimoghaddam, Valentin D. Wegner, Arndt Steube, Elena Gardey, Adrian Feile, Mohamed I. Abdelwahab Hassan, Zoltán Cseresnyés, Andreas Stallmach, Alexander S. Mosig, Marc Thilo Figge, Johannes Stallhofer

**Affiliations:** a Department of Internal Medicine IV, Jena University Hospital, Jena, Germany; b Applied Systems Biology, Leibniz Institute for Natural Product Research and Infection Biology, Hans Knöll Institute (HKI), Jena, Germany; c Faculty of Biological Sciences, Friedrich Schiller University, Jena, Germany; d Cluster of Excellence Balance of the Microverse, Friedrich Schiller University, Jena, Germany; e Institute of Biochemistry II, Center for Sepsis Control and Care, Jena University Hospital, Jena, Germany; f Institute of Microbiology, Faculty of Biological Sciences, Friedrich Schiller University, Jena, Germany; g Facharztpraxis für Gastroenterologie Dr. med. Johannes Stallhofer, Jena, Germany

**Keywords:** Ulcerative colitis, fecal microbiome filtrate, intestine-on-chip, image-based analysis

## Abstract

**Background and aims:**

Ulcerative colitis (UC) is characterized by disruptions of the gut microbiome and an exaggerated mucosal immune response in genetically susceptible individuals. Alterations in the composition of the intestinal metabolome associated with dysbiosis can trigger chronic inflammation. However, it remains unclear whether microbial dysbiosis is the cause or consequence of chronic mucosal inflammation. To address this gap, we aimed to investigate the potential pro-inflammatory effects of sterile fecal microbiome filtrate (FMF) using a microphysiological, immunocompetent intestine-on-chip (IoC) model.

**Methods:**

Sterile FMF from UC patients with active disease (*n* = 6) or in remission (*n* = 4) and non-UC individuals (*n* = 5) were applied to IoC models. Cytokine responses of the epithelial and endothelial compartments were assessed after 24  h, 48  h, and 72  h of incubation, while barrier permeability was evaluated after a period of 72 h. An artificial intelligence-driven image analysis pipeline was developed to quantify structural alterations of the epithelial tissue, including damage and thickness, as well as endothelial and immune cell densities in the IoC model in response to FMF exposure.

**Results:**

FMF from active UC patients significantly increased proinflammatory cytokines (IL-1β, IL-6, IL-8, IL-23, and MCP-1) in the vascular IoC compartment in a time-dependent manner. In contrast, FMF from non-UC or UC patients in remission had no significant impact on the proinflammatory cytokine response compared to untreated media control. Luminal-vascular permeability was increased following the FMF treatment regardless of its origin. Image-based analysis revealed increased epithelial tissue damage and reduced tissue thickness following FMF exposure, alongside decreased endothelial cell density and altered macrophage morphology, independent of UC disease activity.

**Conclusions:**

FMF from UC patients with active disease induces a robust proinflammatory cytokine response in the IoC model, suggesting that UC-associated FMF-derived factors may contribute to the initiation of inflammatory processes relevant to UC pathogenesis. These findings are derived from a simplified intestinal barrier model and require further mechanistic and physiological validation. While image analysis revealed no significant microarchitectural differences among the three FMF groups, the pipelines established standardized metrics to evaluate the impact of FMF-derived factors on intestinal tissue integrity and immune responses, providing a framework for future IoC-based research in UC.

## Graphical abstract

**Figure:**
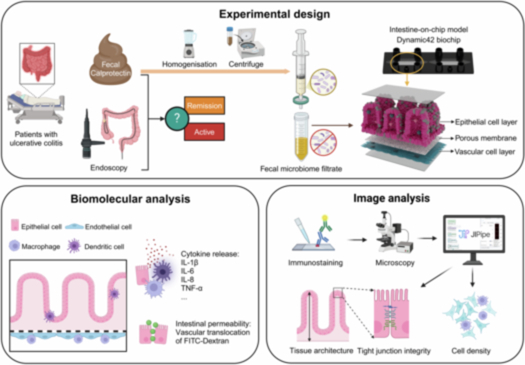

## Introduction

Ulcerative colitis (UC) is a chronic relapsing inflammatory bowel disease (IBD) with a complex and incompletely understood etiology.^1^ It is widely assumed that environmental factors trigger an exaggerated mucosal immune response in genetically susceptible individuals. The gut microbiome has emerged as a key factor in UC pathogenesis, with patients frequently exhibiting microbial dysbiosis characterized by a reduced diversity of beneficial commensals and the expansion of pathogenic bacteria.^2-4^ Whether dysbiosis is a cause or consequence of intestinal inflammation remains unclear. IBD is also associated with altered microbial metabolism, including reduced levels of protective short-chain fatty acids (SCFA)^5^ and secondary bile acids.^6^ These metabolic changes may promote immune cell activation and differentiation, thereby contributing to chronic intestinal inflammation.^7^

Despite the increasing evidence on the relationship between the microbiome and IBD, there is little literature on the role of a sterile fecal filtrate on the intestinal barrier. The publication of Schreiber et al. successfully demonstrated how the transfer of stool filtrate from healthy donors to patients with chronic-relapsing Clostridioides difficile infection (CDI), an intestinal disease that is also associated with colonic inflammation and symptomatically comparable to UC, can reduce inflammation and lead to sustained clinical improvement.^8^ This underlines that disease-modifying effects are mediated by the sterile fecal filtrate – containing various components of the intestinal microbiome, such as proteins, metabolic products such as SCFAs or bile acids, oligonucleotides/DNA, and viruses and virus-like particles (VLPs) such as bacteriophages – rather than by intact bacteria. As the transfer of the fecal microbiome filtrate (FMF) alone was able to alleviate the clinical symptoms of CDI and sufficiently alter the composition of the gastrointestinal microbiota, it suggests that these components of fecal filtrate could mediate the effects of the classical fecal microbial transfer.

Current research highlights the role of microbial metabolites and components, including pathogen-associated molecular patterns (PAMPs), in modulating immune responses and intestinal barrier function.^9^ ^,^ ^10^ As key triggers of immune response, PAMPs contribute significantly to the development of inflammation and tissue damage.^11-13^ These microbiome-derived factors have been implicated in triggering aberrant proinflammatory cytokine production and tissue damage in the colonic mucosa of UC patients.^14^ ^,^ ^15^

**Figure 1.**
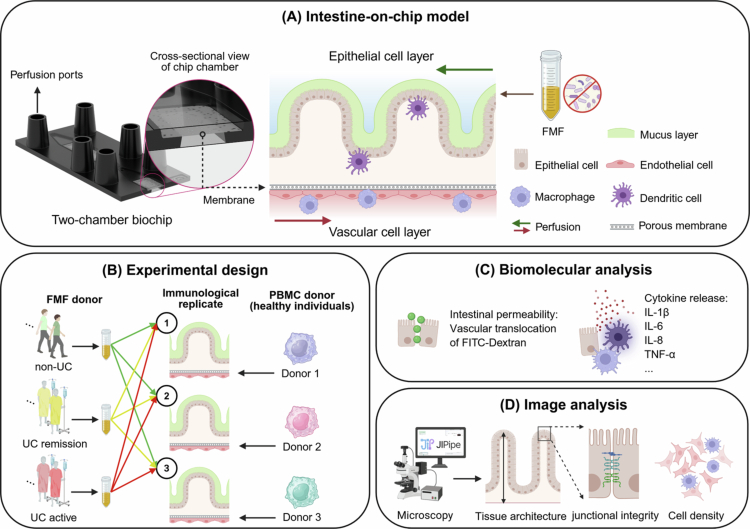
Schematic overview of the IoC experimental workflow integrating FMF. (A) Illustration of the IoC model (left) consisting of two chambers (outlined with a red circle), each containing two compartments separated by a porous membrane. The upper compartment was seeded with intestinal epithelial cells (Caco-2, beige), mimicking the epithelial cell barrier. The lower compartment represents the vascular channel, seeded with endothelial cells (HUVECs, pink), monocyte-derived macrophages (light purple) and dendritic cells (dark purple) that migrate through the membrane into the epithelium. Bidirectional perfusion (green arrow: intestinal; red arrow: vascular) maintains dynamic flow and mimics physiological shear stress and continuous nutrients. FMF from different experimental conditions was introduced into the epithelial compartment. (B) Experimental design. Three independent immunological replicates were established using PBMC-derived macrophages from healthy donors (Donors 1–3). Each PBMC-donor was exposed to sterile FMFs from donors in three clinical groups: non-UC, UC in remission, and UC with active disease, for a total of 15 filtrates. The summarized data per PBMC donor is considered one immunological replicate. (C) Biomolecular analysis, including intestinal permeability and cytokine release. (D) Image-based analysis of microscopic images using JIPipe. The epithelial compartments were analyzed for tissue architecture, including thickness and barrier integrity. Endothelial compartments were assessed by identifying individual cells and quantifying changes in cell density. This figure was created in BioRender.com.

While studies have focused largely on the microbial composition, less is known about the functional properties of the microbial milieu in actively inflamed versus quiescent UC. To address this gap, we employed an immunocompetent intestine-on-chip (IoC) model to study the effects of sterile fecal microbiome filtrate (FMF) derived from UC patients and non-UC individuals. IoC models overcome the limitations of animal models by recreating the intestinal architecture and function within microfluidic devices that operate under dynamic perfusion.^16^ Typically, they feature a two-chamber design with intestinal epithelial cells forming the luminal compartment and a vascular compartment consisting of endothelial cells and immune cells such as monocytes or macrophages.^17-20^ Compared to traditional *in vitro* epithelial cell cultures, the integration of multiple (primarily human) cell types in combination with the optional application of biomechanical stimuli provides higher physiological complexity,^21^ ^,^ ^22^ in which important environmental conditions can be controlled and simulated in a manner comparable to the *in vivo* situation^23^ (Figure 1A).

In recent years, image-based analysis has become increasingly essential for the quantitative evaluation of organ-on-chip models.^24-27^ Here, we established a fully automated image analysis pipeline in JIPipe^28^ that enables compartment-specific analysis in an IoC model. The analysis of epithelial compartments includes the quantification of tissue architecture, such as pixel-based measurements of tissue thickness, as previously introduced,^29^ and the area fraction of tissue damage. In contrast, the analysis of the endothelial compartment focuses on the identification of individual cells, assessing their morphometry, and quantifying cell density, all through employing deep learning algorithms within JIPipe.

In this study, we designed independent immunological replicates using peripheral blood mononuclear cells (PBMC)-derived macrophages from healthy donors. Each replicate of the IoC model was exposed to FMF obtained from individuals in three clinical groups, i.e., non-UC, UC in remission, and UC with active disease (Figure 1B). By combining biomolecular analysis with image-based quantifications, this study aimed to elucidate the inflammatory potential of UC-associated microbial dysbiosis by characterizing the functional and structural consequences of FMF-derived factors on intestinal tissue integrity and immune responses, and establishing standardized metrics for future IoC-based research in IBD (Figure 1C, D).

## Materials and methods

### Study population

Ten adult UC inpatients and outpatients were consecutively recruited between July 2, 2021, and March 31, 2022, at the Department of Medicine IV (Gastroenterology, Hepatology, and Infectious Diseases) of the Jena University Hospital (Jena, Germany). The inclusion criteria for the study were 18 y of age or older and having a confirmed diagnosis of UC based on established endoscopic, histological, and clinical criteria according to the guidelines of the German Society for Gastroenterology, Digestive and Metabolic Diseases.^30^ A recent endoscopic and biochemical assessment of inflammatory activity within 10 d before stool collection was required. Mucosal inflammatory activity was determined using the endoscopic Mayo score (eMAYO) and fecal calprotectin concentrations. Defined cut-off values were used to categorize patients with active disease (eMAYO score ≥ 2 and calprotectin > 250 µg/g) or patients in remission (eMAYO score ≤1 and calprotectin ≤ 250 µg/g). The exclusion criteria included pregnancy and clinical evidence of active infection. Based on these criteria, the patients were categorized into two groups: active UC (6 patients) and UC in remission (4 patients). As a third group, five healthy individuals without acute or chronic infection, without IBD and without a first-degree relative with IBD, and without a known history of acute or other chronic inflammatory, hepatic, or renal disease, and without the use of anti-inflammatory medication, were randomly selected from hospital staff and medical students (non-UC group). Fecal calprotectin levels were not determined in the healthy control group, as concentrations in healthy populations typically show low variability and remain well below thresholds indicative of intestinal inflammation.^31^ ^,^ ^32^ Detailed characteristics of the study population are provided (Supplementary Table S1).

### Sample collection and FMF production

After collection, stool specimens with a stool weight >50 g from UC patients and non-UC individuals were stored in an ultra-low temperature freezer (Eppendorf SE, Hamburg, Germany) at −80 °C until filtration. Before filtration, the thawing process was carried out overnight at 4 °C in the refrigerator. Sterile FMF was prepared from stool using a patented two-stage high-pressure filtration process according to the protocol described by Junca et al.^33^ Briefly, 50 g of stool sample was mixed with 300  mL of PBS (+/+), homogenized for 30  s at 15,000  rpm (Waring™ Laboratory Blender), and filtered (1-mm mesh sieve) to remove coarse particles. The suspension was then divided into 50-mL sample tubes and centrifuged twice at 3,000 g for 15 min. The supernatant was collected (approx. 300 ml) and diluted with PBS (+/+) to 1,000 m for filtration. This was followed by the first filtration (depth filter 15–0.4 µm), followed by sterile filtration (sterile filter 0.22 µm), with the aim of obtaining a sterile, clear filtrate. The final total volume of the sterile filtrate was between 500 and 700  ml.

### Cell culture

The IoC model was built according to Feile et al.^34^ Human umbilical cord vein endothelial cells (HUVEC) were isolated from the human umbilical cord and cultured in endothelial cell growth (EC-) medium.^35^ After reaching subconfluence of 80%, the cells were split and seeded into the chip up to passage 4.

For assembly of the epithelial chip compartment, Caco-2 brush border expressing cells 1 (C2BBe1) (ATCC CRL-2102) were used. These intestinal epithelial cells are derived from human colorectal adenocarcinoma. Owing to their polarized cell arrangement and apical brush border, C2BBe1 cells represent a differentiated, enterocyte-like barrier model of the human colonic epithelium.^36^ C2BBe1 cells were cultured in gut cultivation (GC)-medium (Supplementary Table S2) and harvested at 80% confluence. C2BBe1 cells were used up to passage 35.

### Biochip fabrication and IoC assembly

BC001 biochips (Dynamic42, Jena, Germany), composed of biocompatible polybutylene terephthalate (PBT) and polycarbonate (PC), were used in this study. They contained two culture chambers (upper and lower) separated by a polyethylene terephthalate (PET) membrane (12 µm thickness, 8 µm pore diameter, 1 × 10^5^ pores/cm^2^), providing a cultivation area of 1.1 cm^2^. Each cavity was flushed and perfused independently via inlet and outlet openings (standard Luer ports). The chip served as a platform for the co-culture of endothelial, epithelial, and immune cells,^34^ enabling cellular crosstalk and migration through the porous membrane.^19^

After sterilization of the chip with 70% ethanol for at least 45 min, the endothelial cavity was coated with collagen IV (Supplementary Table S2). HUVEC cells were harvested and seeded with a total of 400,000 cells into the upper (endothelial) cavity. On the following day, PBMCs were isolated from the blood of three healthy donors using density gradient centrifugation (Histopaque-1077; Sigma–Aldrich, Darmstadt, Germany) according to the protocol of Mosig et al.^37^ Isolated PBMCs were cultured in 6-well plates (Greiner Bio-One, Kremsmünster, Austria) of 10 × 10^6^ cells per well in monocyte differentiation medium (Supplementary Table S2) for 24 h at 37 °C. The addition of 10  ng/mL each of the colony-stimulating growth factors M- and GM-CSF (PeproTech, Hamburg, Germany) to the monocyte differentiation medium promoted the differentiation of monocytes into macrophages.^38^ Complete maturation into tissue-resident macrophages took place in co-culture with the endothelial cells in the chip, as previously characterized by cell surface marker analysis demonstrating differentiation of monocytes into two phenotypically distinct subsets resembling mucosal macrophages (CX3CR1+/CD68++) and dendritic cells (CD68+/CD103+).^19^ ^,^ ^24^

Using a lidocaine/EDTA solution (Supplementary Table S2), the monocytes were harvested, and a total number of 100,000 monocytes were seeded on top of the endothelial cavity.

The adherent C2BBe1 cells were harvested, and 500,000 cells were seeded into the lower (epithelial) cavity. The chips were inverted to allow cell attachment to the porous membrane. After another 24 h incubation period, the chips were connected to peristaltic pumps (Ismatec™ Cole-Parmer, Vernon Hills, US) via silicone tubing with an inner diameter of 0.5 mm (Cole-Parmer, Vernon Hills, US), which enabled continuous circular perfusion. The tissue of the chip was supplied with fresh culture media via attached reservoirs with a volume of 3.5  mL. The peristaltic pumps were adjusted to provide a circular bidirectional flow at a rate of 50 µL/min. The biochips were cultivated for 72  h at 37 °C with 5% CO_2_. After 72 h of pre-perfusion, 1 mL of cell culture supernatant was collected from each reservoir and stored at −80 °C until cytokine measurement (timepoint *t* = 0 h). The remaining supernatant was discarded and replaced with 2 mL of fresh endothelial and epithelial perfusion medium. The GC-medium was supplemented with a 1:1 mixture of FMF. The endothelial perfusion medium remained the same. Every 24 h, the supernatants were collected, and the media in the reservoirs were exchanged. After collecting the last cell culture supernatant (*t* = 72 h), a fluorescein isothiocyanate (FITC)-dextran permeability assay was performed to determine the barrier integrity of the IoC model. The tissue layers were then fixed with ice-cold pure methanol and stored at 4 °C until immunofluorescence staining.

### FMF administration

FMF was administered through the epithelial perfusion medium. Depending on the condition, either GC medium alone (standard control) or a 1:1 mixture of GC medium with FMF from non-UC individuals, UC patients in remission, or UC patients with active disease was applied.

### Cytokine measurements

The concentrations of 13 different cytokines and chemokines (IL-1β, IFN-α2, IFN-γ, TNF-α, MCP-1, IL-6, IL-8, IL-10, IL-12p70, IL-17A, IL-18, IL-23 and IL-33) were determined in the endothelial and epithelial perfusion media of the IoC models generated from three independent immunological replicates (three different PBMC donors) using the multi-analyte immunoassay kit LEGENDplex™ Human Inflammation Panel 1 (BioLegend, San Diego, USA) according to the manufacturer’s instructions. The intensity of the fluorescence signal was measured via flow cytometry using BD LSRFortessa™ (BD Biosciences, Franklin Lakes, USA). The device settings and data acquisition recommended by the manufacturer were carried out using BD FACSDiva™ software, and the data were analyzed using the LEGENDplex Data Analysis Software (BioLegend). Samples above the upper detection limit were measured again at a 1:20 dilution with PBS.

### FITC-Dextran permeability assay

To quantify the barrier function of the tissue *in vitro*, a FITC-dextran permeability assay was performed. After 72 h of perfusion, a solution of 1 mg/mL of 4 kDa FITC-dextran in phenol-red-free DMEM was added to the epithelial cavity. After 30 min of incubation, the vascular cell culture supernatants were collected, and the fluorescence intensity of the samples was measured by a Tecan Spark multimode microplate reader (Tecan, Maennedorf, Switzerland). The excitation wavelength was 490 nm, and the emission wavelength was 530 nm. A standard curve in a serial 1:2 dilution was used to convert unitless fluorescence values into concentrations (µg/mL). The detailed compositions of the cell culture media and solutions used in the experiments are provided (Supplementary Table S2).

### Immunofluorescence staining

Indirect immunofluorescence was used to stain various epithelial and endothelial cell components, which were subsequently analyzed microscopically. After the experiment, the cells were fixed with −20 °C cold methanol and stored at 4 °C until staining. The tissue-bearing membrane was cut in half. The front half was used for epithelial staining, and the back half was used for endothelial staining. The membrane pieces were incubated in blocking and permeabilization solution for 30  min at room temperature, before being incubated overnight in a solution of primary antibodies (Supplementary Table S2). Vascular endothelial cadherin (VE-cadherin) and CD68 were stained on the endothelial side, and for the epithelial side, antibodies against E-cadherin and Zonula Occludens-1 (ZO-1) were used. In the subsequent incubation step, the primary antibodies were labeled with fluorescent dye-coupled secondary antibodies. Furthermore, the cell nuclei were stained with DAPI. Cyanine 3 (Cy 3), Alexa Fluor 488 (AF 488), and Alexa Fluor 647 (AF 647) were used as fluorophores (Supplementary Table S3). Before and after each antibody incubation step, the membranes were washed three times with wash solution (Supplementary Table S2) to avoid unspecific binding. Finally, after washing the membrane pieces, they were embedded in fluorescence mounting medium (Agilent Technologies, Santa Clara, USA) on clean glass slides and sealed with a cover slip (Fisher Scientific, Schwerte, Germany). The finished samples were stored at +4 °C until microscopy.

### Image acquisition

3D fluorescence images of AF488-stained ZO-1 and DAPI-stained nuclei in the epithelial compartment, as well as AF647-stained macrophages and DAPI-stained nuclei in the endothelial compartment, were acquired using an LSM 980 confocal laser scanning microscope (Carl Zeiss, Germany). Large field-of-view 3D images were generated through tile scanning and z-stack acquisition. Standard acquisitions consisted of 3 × 3 tiles (9 tiles total), each covering 848.53 × 848.53 µm^2^ at a voxel size of 1 × 1 × 1 µm.^3^ In cases where artifacts were present in the sample, such as air bubbles or other irregularities that could distort image quantification, reduced tiling configurations of 3 tiles (1 × 3) or 6 tiles (2 × 3) were used to minimize artifact interference, covering adjusted total areas from 2375.48 × 848.53 µm^2^ to 2375.48 × 2375.48 µm^2^, respectively. For each sample, one to two images were acquired from non-overlapping areas.

To evaluate how closely the cellular composition within the IoC model reflects the physiological cellular distribution of the human intestinal mucosa, structured illumination microscopy was performed exclusively on untreated biochips using an AxioObserver Z1 microscope equipped with an Apotome (Carl Zeiss, Germany) and a 20 × NA 0.8 air objective. Z-stack images of the nuclei and macrophages were acquired with a field of view of 447.63 × 335.40 µm^2^ and a voxel size of 0.323 × 0.323 × 1 µm^3^ (Supplementary Figure S1).

All the images were recorded at 16-bit depth. The number of z-slices per stack was adjusted according to the tissue thickness to ensure complete coverage of the sample. Brightfield (BF) microscopy was incorporated into the acquisition settings to visualize the membrane pores. The applied excitation wavelengths and emission bandpass filters were as follows: DAPI: 405 nm, 407–506 nm; AF 488: 488 nm, 489–564 nm; and AF 647: 639 nm, 639–734 nm.

### Image preprocessing

To estimate and correct for chromatic aberration in images, a special chip was prepared in which multiply stained (blue/green/orange/red) microspheres were integrated into the epithelial tissue. The 0.1 µm TetraSpeck™ microspheres (Invitrogen) were added at a dilution of 1:100 with PBS -/- after the standard fixation of the chip tissue and incubated for 20 min. The membrane piece was then immediately embedded in mounting medium. Confocal imaging was performed to record several regions of the bead sample at the voxel resolution of the acquired images of 300 × 300 × 200 nm. Subsequently, the chromatic aberration in the bead images was assessed using Huygens Professional software (SVI, Hilversum, Netherlands) and was determined to be less than 1 µm; thus, correction was deemed unnecessary, as it was smaller than the voxel resolution of the images (Supplementary Figure S2).

### Automated analysis of IoC image data in JIPipe

**Figure 2.**
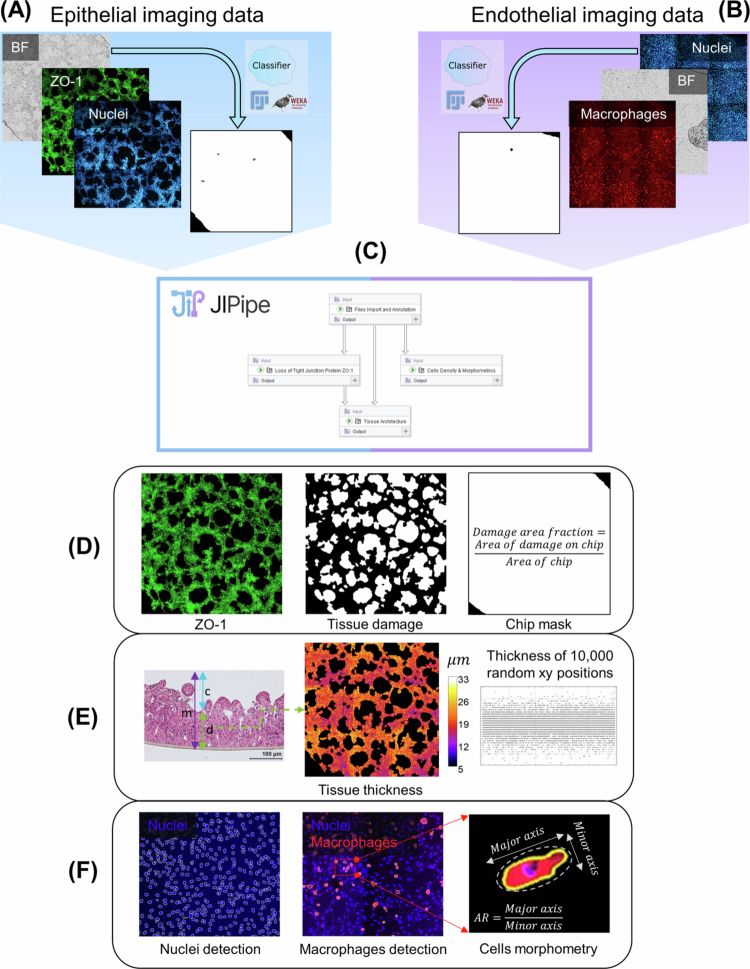
Workflow of automated IoC image analysis in JIPipe. (A) Multi-channel data from the epithelial compartment, including the BF, ZO-1, and nuclei channels. A mask of the chip area is extracted using a trained Weka model applied to the BF channel in Fiji, excluding artifacts such as edges and air bubbles from quantitative analysis. (B) Multi-channel data from the endothelial compartment, including the BF, nuclei, and macrophage channels. A mask of the chip area is extracted using a trained Weka model applied to the nuclei channel in Fiji to prevent interference from artifacts such as edges and epithelial remnants with computational measurements. (C) The JIPipe pipeline is designed to simultaneously analyze images from both epithelial and endothelial cell layers and includes the following analyses: assessment of tight junction integrity based on ZO-1 presence and distribution, quantitative reconstruction of epithelial tissue thickness, and quantification of cell density and morphology in images of the endothelial cell layer. (D) A ZO-1 mask is generated based on intensity thresholds, and the damage area fraction is calculated as the ratio of the total ZO-1 damage area to the total chip area. (E) A heatmap of the tissue structure is obtained by subtracting the interpolated epithelial position (cyan arrow on Caco-2 cell layers^19^) from the membrane position (magenta arrow), producing a heatmap of tissue thickness (green arrow) across all points. The measurements from 10,000 randomly sampled positions represent each image. (F) Nuclei and macrophages are segmented using a deep learning-based model. Morphometric analysis of macrophages includes calculating the area and the AR by dividing the major axis by the minor axis.

To ensure accurate analysis of images from the IoC, a masking technique was implemented to exclude unintended portions of the membrane edges and imaging artifacts captured during tile scanning. For this purpose, the Advanced Weka Segmentation plugin^39^ in Fiji^40^ was trained on representative image features and manual annotations. Post-processing of the predicted masks enabled reliable detection of valid chip areas in images of both epithelial and endothelial cell layers^41^ ^,^ ^42^ (Figure 2A, B, Supplementary Figure S3–S8).

The primary analyses were fully developed in JIPipe.^28^ Multichannel z-stack images were imported and separated into individual channels representing macrophages, ZO-1, nuclei, and brightfield. Three parallel analysis pipelines were established to quantify epithelial tissue damage, epithelial tissue architecture, and endothelial cell characteristics (Figure 2C).

**Epithelial tissue architecture:** Damage to epithelial tight junctions was assessed using the ZO-1 channel. Maximum intensity projections (MIP) of the z-stacks were generated, and low-intensity regions corresponding to disrupted junctions were segmented using a manual threshold. The damage area fraction was calculated by normalizing the tissue damage area to the total chip area (Figure 2D, Supplementary Figure S9). Furthermore, the 3D structure of the tissue was characterized by subtracting the interpolated epithelial tissue position from the membrane position at each x-y coordinate, resulting in a heatmap where each pixel value represents the epithelial tissue thickness at that specific location, as previously described.^29^ Representative measurements were obtained from 10,000 randomly selected positions per thickness heatmap (Figure 2E, Supplementary Figures S10 and S11).

**Endothelial cell density and morphometrics:** To correct stitching artifacts causing duplicated or misaligned cells at tile borders, tiles were manually cropped and recombined prior to analysis (Supplementary Figure S12). Nuclei and macrophages were then segmented using a pretrained CellPose 2.0 model^43^ within JIPipe. The segmented objects were filtered based on intensity and overlap with the chip mask. Morphological features and mean fluorescence intensity (MFI) were extracted for each object, and the cell density was quantified by normalizing the number of detected cells to the chip area (Figure 2F, Supplementary Figures S13 and S14).

### Statistical analysis

Three immunological replicates were generated using PBMC-derived macrophages from healthy donors (*n* = 3). Each replicate was exposed to FMFs obtained from non-UC individuals (*n* = 5), UC patients in remission (*n* = 4), and UC patients with active disease (*n* = 6). In addition, each replicate included 5 media controls without FMF exposure. GraphPad Prism version 8.3.0 (GraphPad Software, San Diego, California, USA) was used to statistically analyze and graphically display the data from the cytokine and permeability measurements. The statistical calculation was performed using the non-parametric Kruskal–Wallis test with Dunn's multiple comparisons test. The results were graphically visualized by column bar graphs with median and interquartile range. Plots of the quantitative analysis of the imaging data were generated using the Seaborn v0.13.2 library, and the statistical analyses were conducted using the Scikit-posthocs v0.10.0 library in Python v3.10. A one-way ANOVA with Tukey’s post-hoc test was used to compare more than two independent samples, with a *p*-value < 0.05 was considered statistically significant. Multiple comparisons are indicated in the figure legends as follows: *p < 0.05, **p < 0.01, ***p < 0.001, ****p < 0.0001. Effect sizes were calculated using Hedges’ g, where a value of g = 0.2 is considered a small effect, g = 0.5 represents a medium effect, and g ≥ 0.8 indicates a large effect.

## Ethical statement

This study was performed following the principles outlined in the Declaration of Helsinki. Written informed consent was obtained from all the subjects before their participation in the study. The study was approved by the local Ethics Committee of the Jena University Hospital as the responsible institutional review board (registration number 2021-2272).

Human peripheral blood was collected from healthy volunteers after receiving written informed consent. The blood donation protocol and use of blood for this study were approved by the Institutional Ethics Committee of the Jena University Hospital (permission number 2207-01/08). HUVECs were collected under ethical approval 2020-1684 and 3939-12/13 after donors provided written, informed consent.

## Results

### Cytokine response

**Figure 3.**
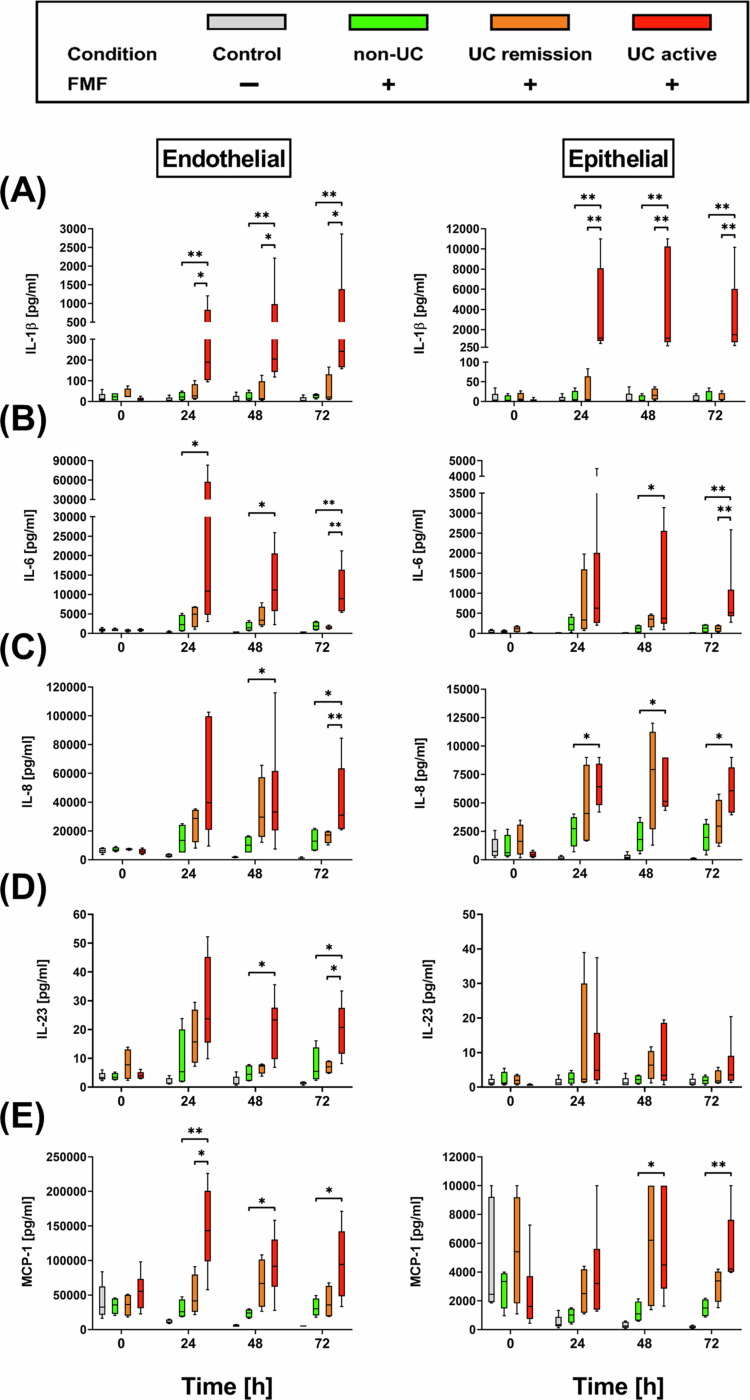
Quantitative analysis of cytokine release. Release of the proinflammatory cytokines (A) IL-1β, (B) IL-6, (C) IL-8, (D) IL-23, and (E) MCP-1 at the endothelial and epithelial sides of the IoC model. The data are shown as box plots with median values ± interquartile range. Statistical significance is denoted as follows: *p < 0.05, **p < 0.01, ***p < 0.001, based on the Mann–Whitney test. Active UC: *n* = 6, UC remission: *n* = 4, non-UC: *n* = 5, and media control: *n* = 5; One representative experiment from three independent immunological replicates is shown.

The concentrations of 13 cytokines and chemokines were quantified in cell culture supernatants from both the endothelial and epithelial compartments. FMF derived from UC patients with active disease induced a markedly stronger increase in the pro-inflammatory cytokines IL-1β, IL-6, IL-8, and MCP-1 on both endothelial and epithelial compartments compared with FMF from non-UC donors or UC patients in remission. Among these, IL-1β showed the most pronounced effect, with significantly elevated levels under active UC conditions across all measured time points. Prior to FMF addition (t = 0 h), all conditions exhibited comparable cytokine levels. The steepest relative increase occurred within the first 24 h, indicating a significant time-dependent rise in cytokine concentrations. With few exceptions, significantly higher cytokine levels (across all cytokines analyzed) were consistently observed in the UC active condition compared with non-UC from 24 h to 72 h. In contrast, the cytokine release did not differ significantly between non-UC and UC remission groups. Compared to the UC active condition, both non-UC and UC remission groups displayed lower variability and a more consistent distribution of measured values. For reasons of clarity, statistical comparisons between the medium control and FMF-stimulated conditions are not shown (Figure 3A–C, E).

Higher IL-23 concentrations were also observed on the endothelial side in the UC active condition compared with the other FMF groups, particularly at 48 h and 72 h. On the epithelial side, IL-23 levels tended to be slightly elevated in both UC remission and UC active conditions relative to non-UC; however, these differences did not reach statistical significance (Figure 3D).

The remaining cytokines measured showed no significant changes between the FMF conditions (Supplementary Tables S4 and S5).

### Intestinal permeability

**Figure 4.**
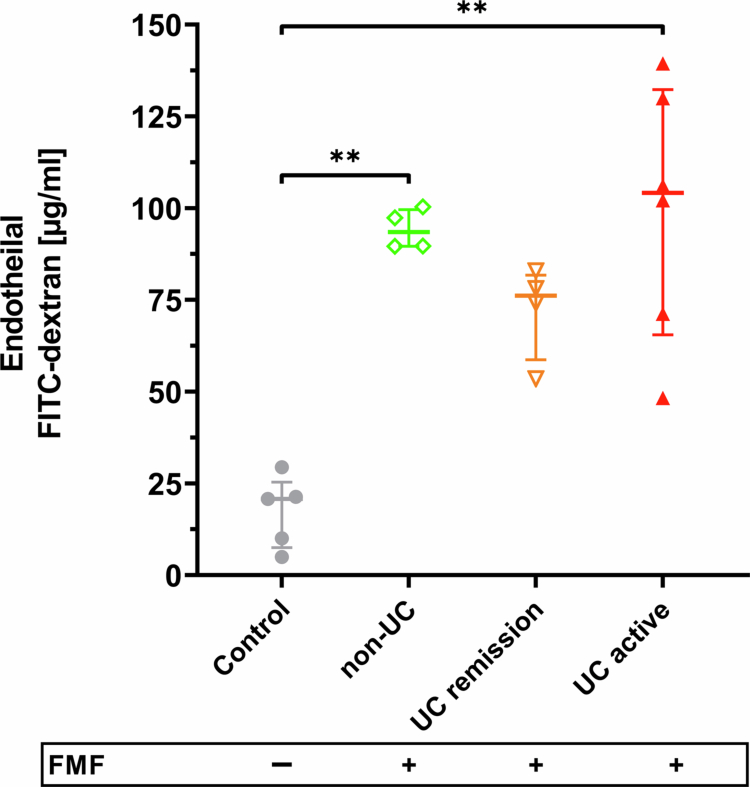
Intestinal permeability for FITC-dextran (4 kDa) after 72 h of stimulation with cell culture medium ± FMF. Visualization of the FITC-dextran concentration measured on the endothelial side of the IoC model. The data are shown as scatter plots with median and interquartile range. Statistical significance is denoted as follows: *p < 0.05, **p < 0.01, ***p < 0.001, based on the Mann‒Whitney test. Active UC: *n* = 6; UC remission: *n* = 4; non-UC: *n* = 5; and media control: *n* = 5, all obtained from a single immunological replicate.

In the pathogenesis of IBD, an increased permeability of the intestinal wall to intraluminal pathogens was postulated.^44^ ^,^ ^45^ Accordingly, our analysis revealed that FMF exposure led to an increase in IoC permeability, as indicated by elevated FITC-dextran levels in the endothelial compartment after 30  min. Relative to the medium control, permeability was increased under all FMF conditions, reaching statistical significance only for FMF from active UC patients and non-UC donors, whereas no significant differences were detected between the FMF groups. Additionally, a marked variability was observed, particularly in the UC active group (Figure 4).

### Epithelial barrier integrity

**Figure 5.**
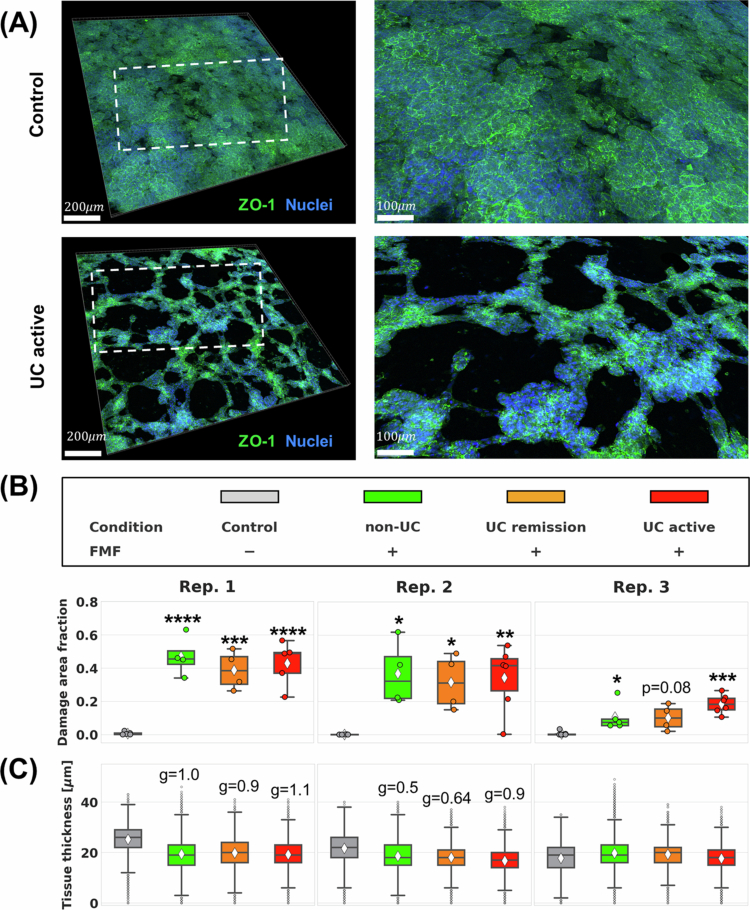
Quantitative analysis of epithelial barrier integrity across replicates. (A) 3D immunofluorescence images of the intestinal epithelial cell layer cultured in DMEM (top) or DMEM supplemented with FMF from UC patients with active disease (bottom). The cells were stained for the tight junction protein ZO-1 (green) and nuclei (DAPI, blue). The left panels show tile scan overviews of the entire epithelial surface (scale bars: 200 µm), while the right panels display magnified views corresponding to the white dashed boxes (scale bars: 100 µm). DMEM-treated samples showed a continuous layer with intact ZO-1 junctional staining, while FMF treatment, independent from IBD status, induces significant disruption of epithelial integrity, characterized by large gaps and discontinuous ZO-1 signals. 3D visualization and rendering were performed using IMARIS 10.2.0 (Bitplane, Switzerland). (B) Damage area fraction per sample, quantified as the total area of ZO-1 protein loss normalized to the chip area. Individual data points shown as circular points overlaid on the box plot correspond to per-image quantification. Statistical significance is denoted as follows: *p < 0.05, **p < 0.01, ***p < 0.001, ****p < 0.0001, based on one-way ANOVA with Tukey’s post-hoc test. (C) Tissue thickness distribution, measured from 10,000 randomly selected points per image, provides a distribution of thickness for each sample. Owing to the large sample size, statistical differences are indicated by Hedges' *g* effect sizes. Effect sizes of *g* ≥ 0.5 are displayed in the figure, corresponding to medium or larger effects (*g* ≥ 0.5: medium; *g* ≥ 0.8: large). Box plots represent the distributions for each group. The horizontal line within each box indicates the median, and the white diamond symbol represents the group mean.

The image analysis results are presented separately for immunological replicates to preserve the inter-donor variability in the IoC model. The analysis of epithelial barrier integrity revealed that FMFs, independent of origin, consistently increased the area fraction of tissue damage relative to the minimal disruption seen in controls (Figure 5A). The UC disease severity (remission or active) did not correspond with an increase in tissue damage. Although a trend towards greater tissue damage was observed in samples treated with FMF from UC patients with active disease compared to those from non-UC individuals or UC patients in remission, this difference reached statistical significance only in one replicate (Figure 5B, Supplementary Figure S15A).

Analysis of the tissue thickness revealed that samples treated with FMF exhibited reduced thickness compared to the control group, a finding confirmed in two of the three replicates. However, tissue thickness was consistent across all FMF treatment groups in each replicate (Figure 5C, Supplementary Figure S15B).

### Endothelial and immune cell densities and morphometrics

**Figure 6.**
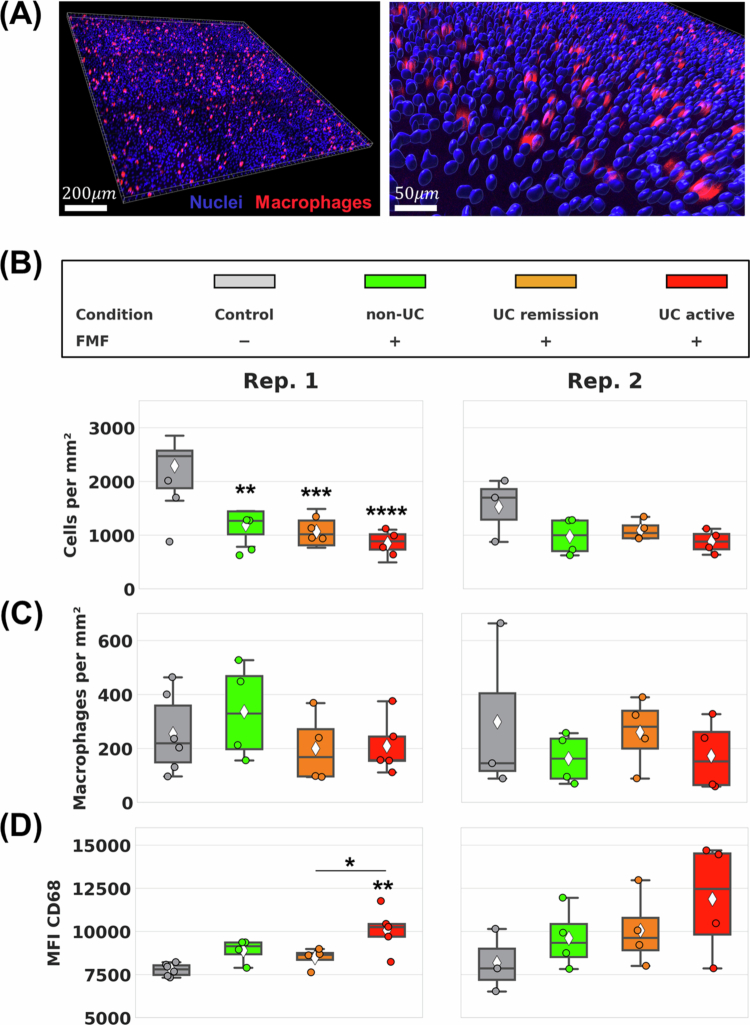
Quantitative analysis of endothelial cell density across replicates. (A) 3D immunofluorescence images of the endothelial cell layer under control conditions. The cells were stained for macrophages (CD68, red) and nuclei (DAPI, blue). Tile scan overview of the entire endothelial surface (left; scale bars: 200 µm) and magnified view(right; scale bars: 50 µm). 3D visualization and rendering were performed using IMARIS 10.2.0 (Bitplane, Switzerland). (B) Endothelial cell density, quantified as the total count of DAPI-stained nuclei normalized by the chip area. (C) Macrophage cell density, calculated as the total count of macrophages normalized by the chip area. (D) The mean fluorescence intensity (MFI) of macrophages was determined as the sum of the fluorescence intensity over the number of macrophage segmentations, normalized by the total number of macrophage pixels. Box plots represent the distributions for each group. Individual data points shown as circular points overlaid on the box plot correspond to per-image quantification. The horizontal line within each box indicates the median, and the white diamond symbol represents the group mean. Statistical significance is indicated by *p < 0.05, **p < 0.01, ***p < 0.001, ****p < 0.0001, based on one-way ANOVA with Tukey’s post-hoc correction.

Further analysis demonstrated a reduction in endothelial cell density in the presence of FMF. A statistically significant decrease compared to the untreated control group was only observed in one replicate (Figure 6A, B, Supplementary Figure S16A). Macrophage cell density remained unaffected across all conditions (Figure 6C, Supplementary Figure S16B). However, the MFI of macrophages displayed an increasing trend correlated with UC severity. In particular, UC active samples exhibited significantly elevated CD68 MFI compared to both non-UC and UC remission groups in one replicate, whereas the other showed a similar but non-significant elevation (Figure 6D, Supplementary Figure S16C).

**Figure 7.**
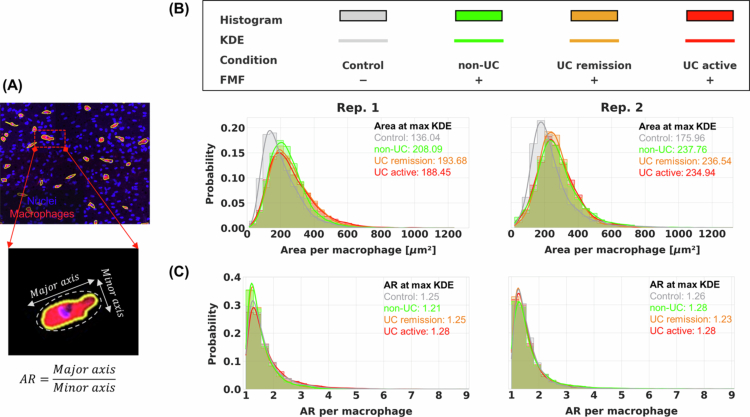
Morphometric analysis of macrophages. (A) Quantification of macrophage AR, calculated as the ratio of the major axis to the minor axis of the fitted ellipsoid, serves as an indicator of macrophage morphology and activity. The macrophages are shown in the red channel, with the DAPI stain in blue. Each segmented macrophage, identified by CellPose, is outlined in yellow. (B) Normalized histogram of the macrophage area, and (C) AR across replicates. The histograms show the distribution of values, with bar heights representing the probability of values within specified intervals, normalized so that the sum of the bar heights equals 1. The kernel density estimate (KDE) provides a smooth, continuous representation of the data distribution using a Gaussian kernel. The values labeled as “Area/AR at max KDE” indicate the macrophage area or AR corresponding to the peak of the density curve, i.e., the most probable morphometric value observed in the dataset.

Furthermore, macrophage shape was quantitatively characterized using two key morphometric parameters: cell area and aspect ratio (AR) as an indicator of the activation status of macrophages^46-48^ (Figure 7A). The normalized histogram of the macrophage cell area revealed notable differences in distribution between the control and FMF-exposed groups. In the control samples, the distributions peaked at 136.04 µm^2^ and 175.96 µm^2^ in biological replicates 1 and 2, respectively, reflecting the most frequent macrophage sizes. In contrast, macrophages exposed to FMF showed a modest rightward shift of approximately 50–70 µm^2^ in the area distribution, with a slight broadening compared to the control group, suggesting the presence of larger, more spread-out cells and increased heterogeneity in cell size, possibly associated with functional activation. Interestingly, different FMF conditions yielded similar area distributions, implying that macrophage size alteration is a general response to FMF exposure and is not strongly influenced by the UC severity. Specifically, the most frequently observed macrophage areas in the FMF-exposed samples clustered around 188.45–208.09 µm^2^ in replicate 1, and around 234.94–237.76 µm^2^ in replicate 2 (Figure 7B). Statistical analysis using Hedges' g effect size revealed only small to medium effect sizes across the comparisons (Supplementary Figure S17A). The normalized histogram of macrophage AR, however, showed no significant changes across conditions, with the AR consistently peaking at a value of approximately 1.2 (Figure 7C, Supplementary Figure S17B).

## Discussion

The gut microbiome and inflammatory disturbances of the intestinal barrier play essential roles in the development of IBD. It is now recognized that IBD is associated with altered intestinal microbial diversity and composition.^4^ As the microbiota as a whole is not experimentally cultivatable and remains largely incompletely characterized, we used a reductionist approach to investigate the impact of UC-associated microbiome-derived factors on intestinal barrier function. Sterile FMFs derived from stool samples of non-UC controls and UC patients with active disease or in remission were applied to a three-dimensional, immunocompetent IoC model to assess their effects on barrier integrity and cytokine responses. Using biochemical analyses, we could demonstrate that FMF from UC patients with active disease can induce a pronounced inflammatory response in this simplified intestinal barrier model, as evidenced by the release of proinflammatory cytokines, whereas FMF increased intestinal permeability regardless of its origin.

It is important to note that the FMF-based stimulation approach used in this study assesses the effects of soluble microbial metabolites, secreted factors, and other bioactive compounds present in sterile fecal filtrates, rather than the effects of living microorganisms or intact microbial communities. While FMFs capture a relevant fraction of the biochemical output of the intestinal microbiome, they do not recapitulate direct microbe‒host cell interactions, quorum-sensing-dependent bacterial behaviors, or the dynamic metabolic activity of a living microbial ecosystem. The responses observed in our IoC model therefore reflect the cumulative impact of the soluble microbiome-derived milieu on intestinal barrier function and innate immune activation, rather than the full spectrum of host‒microbiome interactions occurring *in vivo*.

To capture microstructural tissue alterations induced by FMF exposure, we employed laser scanning confocal microscopy to obtain high-resolution images of both the epithelial and the endothelial compartments of the IoC model. An automated image analysis pipeline^29^ was developed in JIPipe^28^ to quantitatively evaluate multi-channel 3D datasets. This approach enabled detailed characterization of epithelial tissue architecture, including tissue thickness and damage, as well as the assessment of endothelial cell morphology and density. Image analysis revealed that FMF exposure disrupted epithelial integrity across all donor groups, including non-UC, by increasing tissue damage and reducing thickness. These effects occurred independently of UC disease status, as no clear differences were observed between UC remission and active samples. Endothelial cell density was only moderately affected in a replicate-dependent manner, whereas macrophage density remained stable. Nevertheless, macrophages exposed to FMF displayed signs of activation, with UC active samples showing the strongest increase in CD68 intensity and a shift toward larger, more heterogeneous cell shapes. Together, these findings indicate that FMF broadly impairs barrier integrity and modulates macrophage activation, largely irrespective of donor disease status. Given that FMF from healthy control subjects also induced barrier-disrupting effects in the IoC model, it remains unclear whether subclinical intestinal inflammation or alterations in the gut microbiome may have contributed to these observations. Inter-individual variability in microbiome composition is well recognized, and adherence to a healthy lifestyle, including a Mediterranean diet and regular physical activity, has been associated with lower fecal calprotectin levels as well as reduced white blood cell, neutrophil, and lymphocyte counts within the normal range.^49^ Therefore, subclinical inflammatory states capable of affecting intestinal barrier function and inducing measurable cytokine secretion may also have been present among the healthy control subjects. These conditions may have remained undetected, as neither fecal calprotectin nor systemic inflammatory markers were assessed, and dietary habits were not evaluated using standardized questionnaires. Likewise, dietary intake was not assessed in the UC patient cohort.

Notably, while the permeability increase was observed across all patient groups and was not exclusive to UC-derived FMFs, the inflammatory cytokine response, particularly the elevated IL-1β secretion, was more pronounced following exposure to UC-derived filtrates. This dissociation between a relatively conserved barrier response and differential cytokine induction suggests that fecal filtrates from both healthy and diseased donors contain bioactive components capable of modulating epithelial barrier function, which is consistent with the known effects of microbial metabolites such as SCFA and bacterial proteases on tight junction regulation. However, given the relatively high FMF concentration (1:1 FMF/medium) applied in the present study, it cannot be excluded that generalized epithelial or innate immune activation contributed to the observed responses. The 1:1 ratio was chosen to ensure sufficient exposure of the epithelial compartment to FMF-derived bioactive compounds within the 72 h treatment window, yet this concentration may have masked potential dose-dependent differences between the donor groups. The amount of molecules and metabolites (e.g., PAMPs, DAMPs) contained even in FMF from non-UC donors could exceed the capacity of intestinal epithelial cells to maintain homeostasis, ultimately resulting in comparable tissue damage regardless of FMF origin and potentially masking group-specific effects through saturation phenomena. Consequently, the lack of significant differences between UC- and control-derived FMF should be interpreted with caution and does not necessarily indicate biological equivalence between the groups.

While the enhanced IL-1β response induced by UC-derived FMFs suggests that the IoC model may be capable of capturing disease-associated inflammatory differences, the lack of quantification of FMF constituents, such as total protein content or microbial-associated molecular pattern concentrations, limits the interpretation of the underlying mechanisms. In the absence of metabolomic, proteomic, or transcriptomic analyses, it cannot be determined whether the observed effects were driven by qualitative differences in FMF composition between donor groups or by differences in the overall abundance of bioactive compounds. Future studies should therefore incorporate concentration-response analyses with serial FMF dilutions as well as molecular characterization of FMF constituents to provide mechanistic insight into the specific microbial-derived factors, bioactive components, and signaling pathways underlying the differential responses to UC- and non-UC-derived filtrates.

Another factor to consider is that the development of structural tissue damage, such as microerosions and ulcerations, observed in IBD is a time-dependent process.^50^ Consequently, the exposure duration used in the present study (72 h) represents an important experimental parameter that should be considered when interpreting the extent of epithelial barrier disruption and the observed morphological parameters (e.g., cell density, tissue thickness, and damage area). The influence of exposure duration on FMF-induced responses remains to be established and warrants further investigation to determine whether prolonged exposure results in more or less pronounced alterations in barrier integrity and inflammatory signaling.

The central question regarding which exact components are responsible for the pro-inflammatory effects of the filtrate remains unanswered. The metabolites produced by the intestinal microbiome, such as secondary bile acids or SCFA, have a significant influence on immunological function and the integrity of the intestinal barrier.^14^ ^,^ ^51^ Given the anti-inflammatory properties of SCFA and secondary bile acids, it can be assumed that FMF from UC patients contains lower levels of these metabolites compared to non-UC donors. This reduction is due to the decreased abundance of oligosaccharide-fermenting and bile acid-converting bacteria related to intestinal dysbiosis. Future studies should include the quantification of these bacterial metabolites in the stool and FMF of UC patients in combination with supplementation experiments to determine the clinical relevance of these metabolites for maintaining intestinal immune homeostasis. Previous studies have already described alterations in the microbial metabolome in IBD patients. A recent publication investigated colonic luminal fluid samples from UC patients and non-UC individuals to identify factors with pro-inflammatory potential. In particular, the elevated concentration of IgA-coated bacterial extracellular vesicles in the samples of active UC patients proved to be potent activators of pro-inflammatory responses in CD89+- (IgA-receptor) immune cells, triggering a pro-inflammatory cytokine release with significantly increased IL-6 and IL-8 levels and exacerbating intestinal inflammation in a dextran sodium sulfate-induced colitis mouse model.^52^

In addition to alterations in the intestinal metabolome or bacterial vesicles, cytokines may be potential mediators of the immunomodulatory effect of FMF. Proinflammatory cytokines, including IL-1β, IL-6, TNF-α, and many others, are key mediators of inflammation. These proteins are primarily secreted by monocytes and macrophages, and to a lesser extent by endothelial or epithelial cells. UC patients have elevated concentrations of IL-1β in the inflamed mucosa as well as in the stool, which mainly originates from activated mucosal macrophages.^53^ ^,^ ^54^

IL-1β triggers an inflammatory cascade that leads to the stimulation of macrophages and consequently to the release of further cytokines on the endothelial side. Several studies indicate that IL-1β impairs the function of the epithelial tight junction barrier and thus contributes to increased intestinal permeability.^55^ ^,^ ^56^

Recent investigations have emphasized the pivotal contribution of IL-23 to the pathogenesis of inflammatory bowel diseases. IL-23 is a proinflammatory heterodimer of the IL-12 cytokine family consisting of the IL-12p40 subunit and the unique IL-23p19 subunit. It is primarily secreted by antigen-presenting cells and macrophages in gastrointestinal tissues.^57^ Through its receptor (IL-23R), which is expressed on various innate and adaptive immune cells, IL-23 regulates their activation and cytokine production. The targeting of IL-23 and its downstream IL-23R signaling has become a central strategy in controlling gut inflammation.^58^ Recent evidence indicates that several IL23p19-specific neutralizing monoclonal antibodies, including risankizumab and mirikizumab, demonstrate both efficacy and safety in the treatment of patients with UC.^59^

While our IoC model does not fully recapitulate the complexity of the human intestinal mucosa, it integrates several key architectural and functional features relevant to intestinal physiology. The co-culture of epithelial, endothelial, and immune cells under dynamic flow conditions enables the formation of villus-like and crypt-like epithelial structures, the establishment of a functional endothelial barrier, and the incorporation of tissue-resident immune cells. Importantly, although the initial cell seeding numbers do not directly reflect the cellular composition of the intestinal wall, the distinct proliferative behaviors of the individual cell types lead to a progressive shift in the cellular ratios over the culture period. C2BBe1 cells proliferate three-dimensionally under flow, forming multi-layered, villus-like and crypt-like folding of the epithelial barrier.^19^ ^,^ ^60^ ^,^ ^61^ This engineered folding arises from the flow-dependent removal of the basolaterally secreted Wnt antagonist DKK-1 and a flow-induced increase in FZD9 expression rather than from a functional stem cell niche, and it does not establish authentic crypt-villus compartmentalization or lineage diversity. Further, HUVECs reach confluency as a contact-inhibited monolayer, and monocyte-derived macrophages do not undergo further division after seeding. Consequently, at the time of FMF application, epithelial cells substantially outnumber endothelial and myeloid cells. This finding is supported by our quantitative image analysis, which revealed a pronounced epithelial dominance of 82.9%, compared to 14.8% for endothelial cells and 2.2% for macrophages (Supplementary Figure S1). These cellular distributions align with the hierarchy observed in human intestinal tissue, where epithelial cells constitute approximately 70%, endothelial cells constitute ~5%, and myeloid cells constitute ~3% of the total cell population.^62^

Several limitations of the current model should be acknowledged. The use of cell lines (C2BBe1) and non-intestinal primary cells (HUVECs) rather than patient-derived intestinal organoids and tissue-specific endothelium represents a simplification that limits direct translational conclusions. As a clonal, adenocarcinoma-derived line, C2BBe1 cells exhibit a constitutively active Wnt/β-catenin signaling state and a comparatively uniform, tight epithelial barrier, and they lack the stem, goblet, Paneth, enteroendocrine, tuft, and M cell populations as well as the spatial morphogen gradients of the native crypt-villus axis. The villus-like and crypt-like structures observed under flow, therefore, represent engineered epithelial folding rather than bona fide crypt-villus architecture.^60^ ^,^ ^61^ This limited epithelial diversity constrains the dynamic epithelial‒immune crosstalk in modeling IBD pathogenesis. Furthermore, our model lacks key components of the intestinal immune system, including adaptive immune cells, innate lymphoid cells, and the enteric nervous system. The absence of a luminal microbiome and the use of sterile filtrates rather than live microbial communities preclude the study of direct host‒microbe interactions. Future iterations of this platform may incorporate iPSC-derived intestinal cell types and a broader immune cell repertoire to further enhance physiological relevance.

The biological variability observed between individual FMF donors is an inherent feature of working with patient-derived samples, where each filtrate represents a unique composition of microbial metabolites and signaling molecules reflecting the donor-specific microbiome. Such variability is also reflected in immune cell responses. In our design, this variability originates from two independent factors. The host immune factor is represented by the three immunological replicates from different PBMC-derived macrophage donors, and the microbial stimulus, represented by the individual FMF donors within each clinical group. We controlled for host variability by exposing all four conditions in parallel within each immunological replicate, so that condition-dependent comparisons remained internally matched, and we reported the image-based readouts per replicate rather than pooling them to preserve both compartments of variability. The disease-associated response we resolve is carried out by the innate immune cell source and is structured across the two chambers of the model. Within the three immunological replicates analyzed for cytokines, the proinflammatory signature, most consistently IL-1β, separated active UC from non-UC and remission filtrates despite the inter-individual variability among the FMF donors, and IL-23 was elevated on the vascular side under active UC conditions, while the epithelial difference did not reach significance. Across independent immunological replicates, the macrophage CD68 intensity increased with UC severity, reaching significance in one replicate and showing a concordant trend in the other. A signal that separates active UC from controls despite this biological variability is more informative than one obtained from pooled stimuli, in which individual differences are averaged out. By contrast, the generalized increase in barrier permeability and the microarchitectural changes were comparable across all FMF groups, which is consistent with the concentration-driven saturating effect. The ability of the IoC platform to resolve these inter-individual differences may represent a relevant advantage over approaches that rely on pooled or standardized stimuli. Given the well-documented disease heterogeneity and variable treatment responses in IBD,^63^ systems capable of capturing donor-specific response patterns could be valuable for functional analysis of microbiome‒host interactions. While the present study was not designed to systematically evaluate the diagnostic or stratification potential of such individualized readouts, and the technically demanding and time-intensive nature of chip assembly limited the number of biological donors included, the observed donor-dependent differences in cytokine release and barrier responses suggest that this direction warrants further investigation in larger patient cohorts.

The combination of an IoC model and the FMF of UC patients shows the indirect effect of intestinal microbial factors on the intestinal barrier. Whether the barrier-damaging effect of FMF results from an excess of pro-inflammatory factors, a deficiency of protective factors, or a combination of both remains unresolved. Future studies will therefore need to identify not only the specific pro-inflammatory substances present in the filtrate, but also the protective factors that contribute to the maintenance and homeostasis of the intestinal barrier and can be influenced therapeutically. The impressive clinical success of fecal microbial transfer in recurrent *CDI* shows that restoring eubiosis could be a key target in the prevention and treatment of IBD.^64^ However, despite promising case reports for UC^65^ and positive data on fecal microbial transfer in the murine Dextran sulfate sodium (DSS-) colitis model,^66^ this therapeutic concept has not yet been established for IBD, in contrast to recurrent *C. difficile* enteritis.^67^

Moreover, a key advantage of our image analysis pipeline is its adaptability, not only to diverse organ-on-chip models but also to different cellular compositions within these systems. The JIPipe platform allows for straightforward extension to incorporate additional analysis readouts such as immune cell infiltration, microbial community dynamics, microbiome‒host interactions, and the spatial localization of inflammatory markers. In conclusion, the combination of the IoC model and our JIPipe-based analysis pipeline represents a promising tool not only for studying IBD and evaluating the severity of IBD status, but also for advancing the development of novel therapeutic strategies.

## Supplementary Material

SupplementaryMaterial_Revision2.pdf SupplementaryMaterial_Revision2.pdf

## Data Availability

The data underlying this article are available at https://asbdata.hki-jena.de/Schaal_AkbarimoghaddamEtAl2025_FMF.

## Conference presentation

Parts of the work were presented at *Viszeralmedizin*, Hamburg, 2023.

## Abbreviations

IBD: Inflammatory bowel disease
IoC: Intestine-on-chip
UC: ulcerative colitis
FMF: Fecal microbiome filtrate
FITC: Fluorescein isothiocyanate
ULT: Ultra-Low temperature
HUVEC: Human umbilical cord vein endothelial cells
C2BBe1: Caco-2 brush border expressing cells 1
PBMC: Peripheral blood mononuclear cells
GM-CSF: Granulocyte-macrophage colony-stimulating factor
M-CSF: Macrophage colony-stimulating factor
PBS-/: Phosphate-buffered saline without calcium and magnesium
PBS+/+: Phosphate-buffered saline with calcium and magnesium
IL: Interleukin
IFN: Interferon
TNF: Tumor necrosis factor
MCP: Monocyte chemotactic protein
EDTA: Ethylenediaminetetraacetate
CDI: Clostridioides difficile infection
LCA: lithocholic acid
SCFA: short chain fatty acids
PAMP: Pathogen-associated molecular pattern
DAMP: Damage-associated molecular pattern
MIP: Maximum intensity projection
MFI: Mean fluorescence intensity
JIPipe: Java Image Processing Pipeline
