# Supplementary material for: Assessing the proinflammatory potential of sterile fecal microbiome filtrate from ulcerative colitis patients using an intestine-on-chip platform and automated image analysis
Source: Gut Microbes. 2026 Jul 28;18(1):2701400. doi: 10.1080/19490976.2026.2701400 (PMC13432893; doi:10.1080/19490976.2026.2701400)
Supplement: SupplementaryMaterial_Revision2.pdf [file KGMI_A_2701400_SM4559.pdf]

Supplementary material for

Assessing the Proinflammatory Potential of Sterile Fecal Microbiome Filtrate from Ulcerative Colitis Patients Using an Intestine-on-Chip Platform and Automated Image Analysis

Tobias Schaal[1, *], Parastoo Akbarimoghaddam[2,3,4 *], Valentin D. Wegner[4,5], Arndt Steube[1], Elena Gardey[1], Adrian Feile[4,5], Mohamed I Abdelwahab Hassan[4,5], Zoltán Cseresnyés[2], Andreas Stallmach[1], Alexander S. Mosig[4,5], Marc Thilo Figge[2,4,6, #], Johannes Stallhofer[1,7 #]

[1] Department of Internal Medicine IV, Jena University Hospital, Jena, Germany

[2] Applied Systems Biology, Leibniz Institute for Natural Product Research and Infection Biology, Hans Knöll Institute (HKI), Jena, Germany

[3] Faculty of Biological Sciences, Friedrich Schiller University, Jena, Germany

[4] Cluster of Excellence Balance of the Microverse, Friedrich Schiller University, Jena, Germany

[5] Institute of Biochemistry II, Center for Sepsis Control and Care, Jena University Hospital, Jena, Germany

[6] Institute of Microbiology, Faculty of Biological Sciences, Friedrich Schiller University, Jena, Germany

[7] Facharztpraxis für Gastroenterologie Dr. med. Johannes Stallhofer, Jena, Germany

* These authors contributed equally to this work.

Short title

FMF from UC patients in an intestine-on-chip

[#] Correspondence to:

Marc Thilo Figge, Prof. Dr.
Applied Systems Biology
Leibniz Institute for Natural Product Research and Infection Biology – Hans Knöll Institute
Beutenbergstraße 11a
07745 Jena, Germany
Telephone: +49 3641 532-1416
Fax: +49 3641 532 2416
E-Mail: thilo.figge@leibniz-hki.de

Johannes Stallhofer, Dr.
Facharztpraxis für Gastroenterologie
Dr. med. Johannes Stallhofer
Westbahnhofstraße 2
07745 Jena, Germany
Telephone: +49-3641-622180
Fax: +49-3641-622181
E-Mail: mail@facharztpraxis-gastroenterologie.de

Evaluation of cellular composition within the IoC model

Quantitative image analysis to evaluate cellular composition within the intestine-on-chip (IoC) model, was conducted using IMARIS 11.0.1 (Bitplane, Switzerland). DAPI-stained nuclei were detected using the “Spots” module with an estimated XY diameter of 8 µm. CD68-stained macrophages were segmented using the “Surfaces” module. Prior to segmentation, images were smoothed with a surface grain size of 2 pixels, followed by background subtraction, with the diameter of the largest sphere set to 30 µm. Subsequently, manual thresholding was applied to identify foreground signal. To minimize false-positive detections, objects in all channels were filtered based on mean intensity and voxel count. When z-stacks included both compartments, a z-position filter was applied to exclude signals from the unwanted region (**Supplementary Fig. S1A-B**). The total number of DAPI-stained nuclei was used as a measure of total cell count in both epithelial and endothelial compartments. In the endothelial compartment, macrophage counts (CD68-positive) were subtracted from the total DAPI count to estimate the number of endothelial cells (**Supplementary Fig. S1C**).

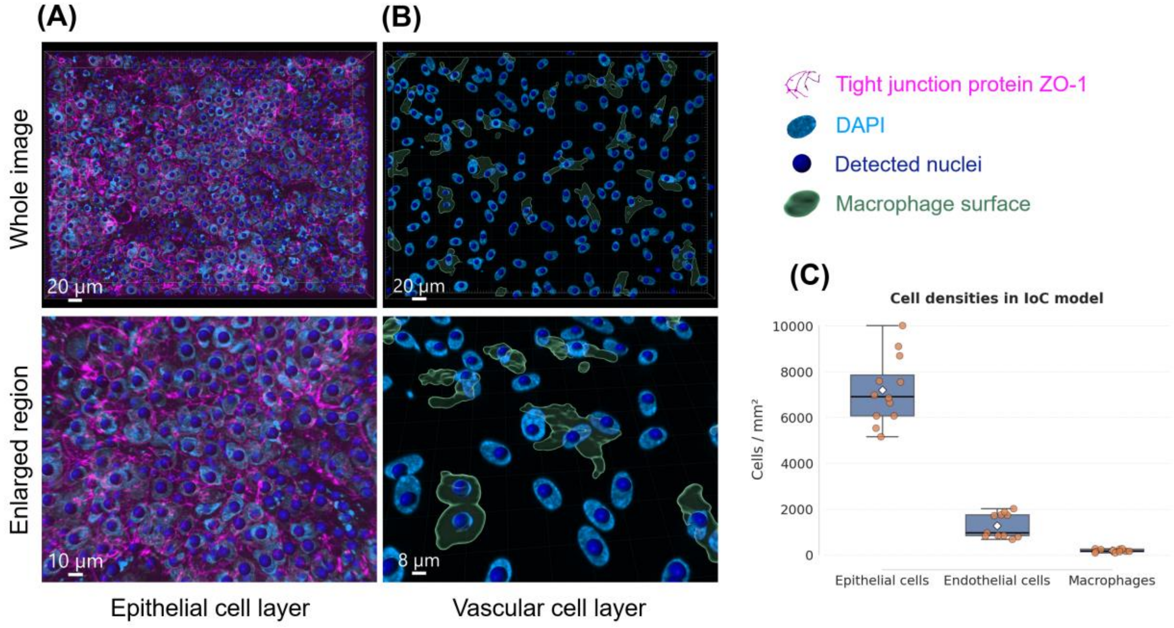

Supplementary Figure S1. Quantification of cellular composition in the IoC model. (A) Immunofluorescence images of the epithelial and **(B)** vascular cell layer. E-cadherin (magenta), nuclei (cyan), detected nuclei (blue spheres), and segmented macrophages (green) are visualized. First row, whole images; second row, corresponding enlarged views. **(C)** Quantification of cell densities (cells/mm²) in the IoC model. Box plots show the distribution of each cell type, with the horizontal line indicating the median, individual data points overlaid as orange circles, and the mean represented by a white diamond. Data were derived from 10-12 images per epithelial and endothelial compartment across two biological experiments.

Quantitative image analysis of cell densities in the IoC model revealed a predominance of epithelial cells, with a mean density of 7195.74 cells/mm², accounting for ~82.9% of the total cell population, followed by endothelial cells (1286.72 cells/mm²; ~14.8%) and macrophages (194.37 cells/mm²; ~2.2%). Detailed distribution metrics are provided below.

Cell type	Mean (cells/mm²)	% of total	Median	Q1	Q3	IQR
**Epithelial**	7195.74	82.9%	6923.76	6079.52	7869.58	1790.05
**Endothelial**	1286.72	14.8%	972.46	842.57	1755.09	912.51
**Macrophages**	194.37	2.2%	166.52	159.86	259.77	99.91

Importantly, the resulting cellular distribution approximates the hierarchical organization of the human intestinal mucosa, in which epithelial cells represent the major fraction (~70%), while endothelial (~5%) and myeloid (~3%) populations remain comparatively minor[1].

These findings support that, despite simplified cell sourcing and non-physiological seeding ratios, the IoC model dynamically evolves toward a physiologically relevant cellular composition. These proportions reflect the distinct proliferative behaviors of the incorporated cell types. While monocyte-derived macrophages do not proliferate after seeding and HUVECs form a contact-inhibited monolayer, C2BBe1 epithelial cells undergo continuous three-dimensional expansion under flow conditions, forming crypt- and villus-like structures. This results in a progressive shift in cellular composition over time, with epithelial cells becoming the dominant population.

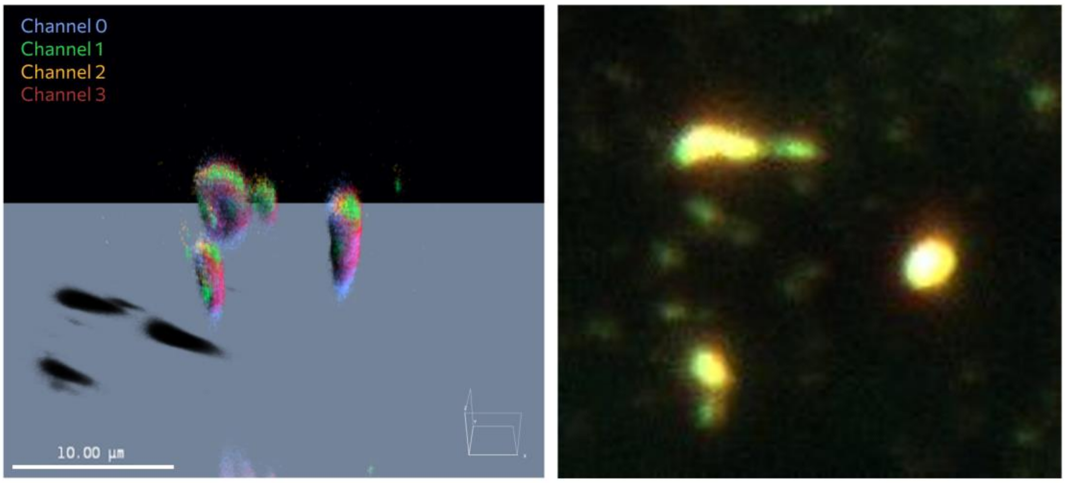

(A) 3D view **(B)** XY view

Supplementary Figure S2. Assessment of chromatic aberration in multi-channel fluorescence beads imaging. (A) Huygens Professional (SVI, Hilversum, The Netherlands) Simulated Fluorescence Process (SFP) Volume Renderer. The 3D view illustrates that the green, orange, and red channels exhibit minimal shifts (below 1 µm) relative to the blue channel. **(B)** Huygens Maximum Intensity Projection (MIP) Renderer. The XY view demonstrates that the beads are nearly perfectly aligned.

Weka-based mask extraction for analysis of epithelial cell layers

Artifacts in the IoC microscopy images arise due to several factors, particularly when using tile scan setups to capture larger fields of view. Tile scanning can inadvertently include portions of the membrane edge, which are not relevant to the analysis. Additionally, transferring samples from the chip chamber to a glass slide often introduces air bubbles, which are especially prone to appear in areas of tissue loss due to the presence of fecal microbiome filtrate (FMF), occupying the vacant spaces (**Supplementary Fig. S3**). These bubbles interfere with the detection of membrane pores in the brightfield (BF) channel.

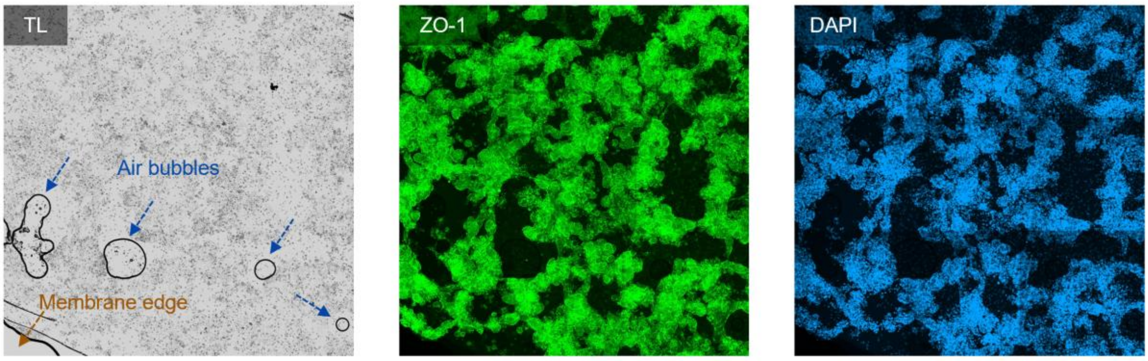

Supplementary Figure S3. Common artifacts in intestine-on-chip microscopy. Tile scanning can capture unintended portions of the membrane edge (indicated by orange arrow), while air bubbles, which appear as thick dark lines in the acquired BF images, are often introduced during sample transfer from the chip chamber to the glass slide (indicated by blue arrows). These bubbles tend to accumulate in areas of tissue loss, as seen in comparison with the ZO-1 and DAPI channels, hindering accurate image analysis.

Classifier Training: To define a mask, the *Advanced Weka Segmentation* plugin[2] in Fiji[3] was used. Masks were generated based on the BF images of both epithelial and endothelial cell layer. A Minimum Intensity Projection was applied to generate a 2D dataset comprising 155 images, of which 50 contained artifacts. From these, a subset of 20 images was randomly selected for classifier training. The images were subsequently down sampled by a factor of 0.3 using bilinear interpolation via the *Scale* command in Fiji to optimize training speed. Both the edges of the membrane and air bubbles were observed to appear as sharp, dark lines in the BF images. Therefore, for classifier training, two classes were defined: "Chip", representing all areas excluding artifacts, and "Artifacts", referring to the sharp dark lines. At each iteration, portions of these classes were manually annotated using the freehand selection tool in Fiji. The labeled data was then used to train the classifier. The default training features

were selected, including Gaussian blur, Hessian, membrane projections, Sobel filter, and Difference of Gaussians (DoG). For the membrane patch size, a value of 19 was used, with a minimum sigma of 1 and a maximum sigma of 16. The classifier model used was Fast-Random Forest (RF) with the following default settings: NumTrees = 200, NumThreads = 36, MaxDepth = unlimited, NumFeatures = 2, and NumDecimalPlaces = 2. In the first iteration, the trained classifier was saved to a file in the standard Weka format (.model), and the trace information was stored in a separate data file (.arff). For subsequent iterations, the newly imported image, along with the previously saved classifier and data file, were loaded. The two classes were then manually annotated again, and the classifier was retrained (**Supplementary Fig. S4**).

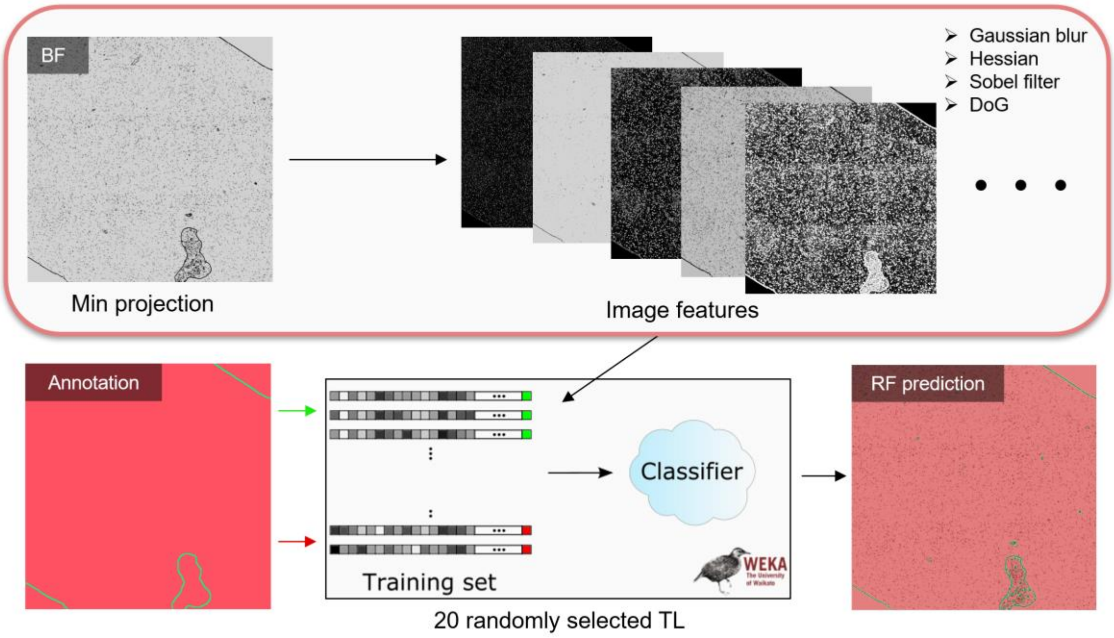

Supplementary Figure S4. Weka-based mask extraction for image analysis of epithelial cell layer. Minimum intensity projections of BF images were manually annotated into two classes: "Chip", representing all non-artifact areas, and "Artifacts", representing sharp dark lines. Various image features, including Gaussian blur, Hessian, Sobel filter, and DoG, along with the annotations, are put into the RF classifier in *Advanced Weka Segmentation* plugin in Fiji for training on a set of 20 images. The trained model is subsequently used to predict artifact masks for the full epithelial dataset.

Classifier prediction and post processing: The final trained model was applied on all 2D BF images of epithelial cell layer to obtain the probability map of the class “Artifacts” (**Supplementary Fig. S5A**). A threshold of 0.5 was set to this probability map to obtain a

binary mask of artifacts (**Supplementary Fig. S5B**). Then dilation with an octagon-shaped structuring element with a radius of 1 pixel was applied using the *MorphoLibJ*[4] plugin to reconnect potentially interrupted or disconnected lines (**Supplementary Fig. S5C**). The Particle Analyzer tool from the *BoneJ*[5] plugin was applied to exclude objects with an area smaller than 1000 µm², ensuring that only sufficiently large artifacts remained. These artifacts could include air bubbles, membrane edges, and small dark regions from where pores could not be accurately detected (**Supplementary Fig. S5D**). The *Fill Holes* command in Fiji was used to fill regions corresponding to air bubble artifacts (**Supplementary Fig. S5E**). This process ensures the exclusion of regions associated with air bubbles on the chip. To accurately exclude edge artifacts, the previous image (**Supplementary Fig. S5E**) was inverted (**Supplementary Fig. S5F**). Triangular objects touching the edges were then identified using *Particle Analyzer* from the *BoneJ*[5] plugin with a maximum area threshold of 10^6 µm², followed by *Kill Borders* command from *MorphoLibJ*[4] plugin allowing us to isolate edge-touching components without including the larger areas associated with the chip itself (**Supplementary Fig. S5G**). Finally, the detected objects were binarized and combined with the results from the "Filling holes" step. The combined output was then inverted and up-sampled to the original image resolution, thereby generating the final chip mask (**Supplementary Fig. S5H**).

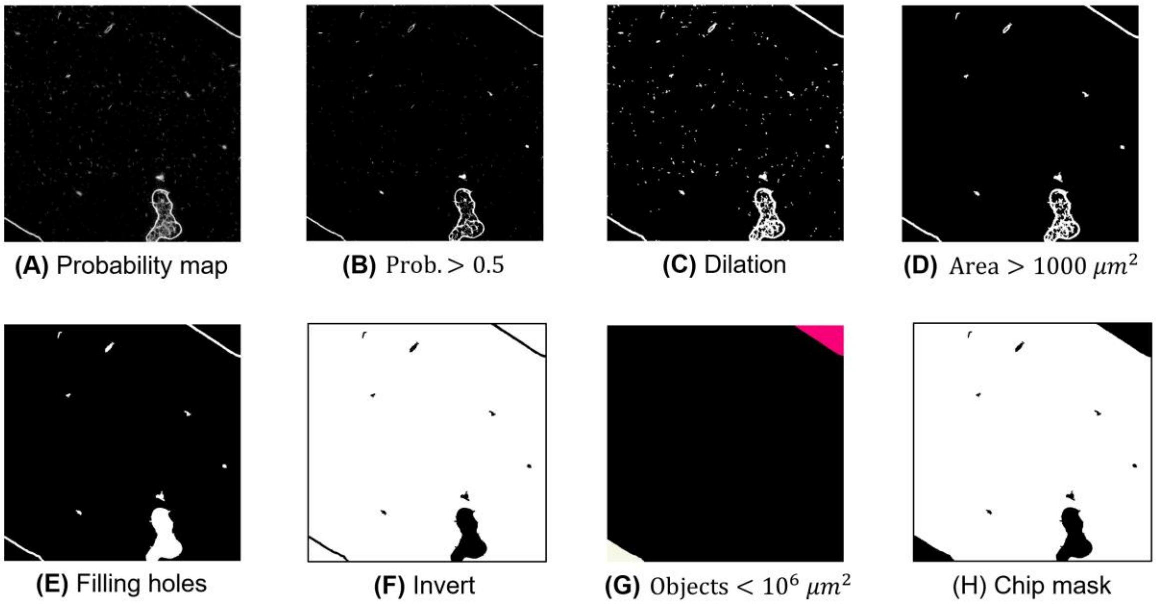

Supplementary Figure S5. Post-processing workflow for epithelial data masks. (A) Probability map of artifacts as predicted by the trained Weka model. **(B)** Binary artifact mask generated by applying a probability threshold of 0.5. **(C)** Dilated mask using an octagonal structuring element with a radius of 1 pixel. **(D)** Retained objects with an area greater than 1000 µm², representing actual artifacts. **(E)** *Fill Holes* applied to fill air bubble regions. **(F)**

Inverted mask to prepare for final processing. **(G)** Detection of edge-touching objects. **(H)** Final mask of the chip by combining (E) and (G), isolating relevant regions and excluding artifacts.

Mask customization based on intended readouts

For evaluating tissue architecture, both the DAPI and BF channels are required, with the BF channel (**Supplementary Fig. S6A**) used to estimate membrane positioning. In this case, the mask must exclude both edge artifacts and air bubbles to ensure accurate assessment (**Supplementary Fig. S6B**). In contrast, for quantifying the loss of the tight junction protein ZO-1, only the ZO-1 channel is used. In this case, it is sufficient to exclude only the membrane edges to avoid misinterpreting them as tissue damage. To generate this mask, the *Fill Holes* command was applied to all previously predicted masks (**Supplementary Fig. S5H**). This process excluded the membrane edges while filling in other artifacts, such as air bubbles. In cases where air bubbles were located along the edges, manual corrections were made to ensure accuracy (**Supplementary Fig. S6C**).

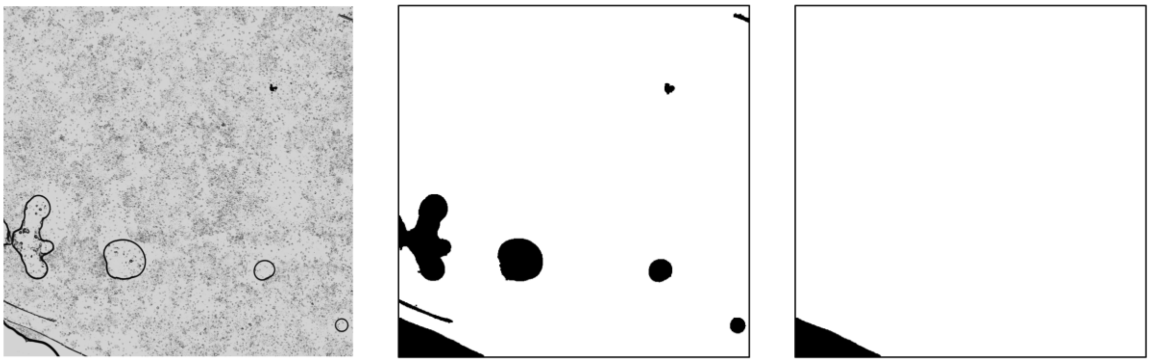

(A) BF **(B)** Mask for Tissue Architecture **(C)** Mask for ZO-1 loss

Supplementary Figure S6. Readout-dependent masks for epithelial image analysis. (A) Minimum intensity projection of BF channel. **(B)** Mask for tissue architecture: For tissue architecture analysis, the BF channel is used to determine pore positions, requiring exclusion of both membrane edges and air bubble artifacts. **(C)** Mask for ZO-1 loss: To quantify ZO-1 loss, only membrane edges need to be excluded.

Weka-based mask extraction for analysis of endothelial layers

In the image analysis of endothelial cell layer, in addition to membrane edges, a different category of artifacts was also observed. These artifacts include large, overlapping regions, which are epithelial remnants from the opposite side of the membrane that were not completely removed during sample preparation. DAPI staining contributed to these artifacts by marking nuclei on both the endothelial and epithelial sides, producing non-specific signals. Furthermore, the dye AF 647, used for macrophage staining on the endothelial side, also bound to the Ki-67 proliferation marker on the epithelial side. As a result, these residual areas introduced interfering signals in both the endothelial cell nuclei and macrophage channels. These artifacts were unavoidable due to several factors: the epithelial remnants could not be fully removed during preparation without risking damage to the sample, and the tile-scanning approach used for larger fields of view inevitably captured these regions in the images (**Supplementary Fig. S7**).

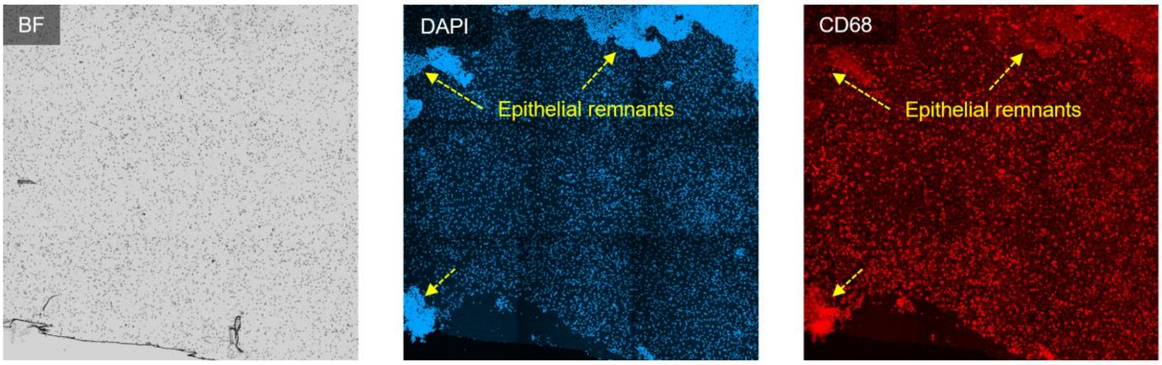

Supplementary Figure S7. Artifacts in images of endothelial cell layer. Yellow arrows indicate epithelial remnants from the opposite membrane side, which could not be fully removed during sample preparation, resulting in unavoidable background signals in the image.

The procedure for obtaining the endothelial mask followed a similar approach to that used for epithelial mask extraction. The *Advanced Weka Segmentation*[2] plugin was employed to define the masks, utilizing the DAPI channel from a set of 63 nine-tile images of endothelial tissue. An MIP was first applied to generate a 2D dataset. From this dataset, five images exhibiting epithelial remnant artifacts were selected for classifier training. Two distinct classes were defined: "Chip", representing nuclei regions, and "Artifacts", which included epithelial remnants. The training configuration, including the selected model and its parameters, were identical with those used in the epithelial mask extraction process.

The final trained model was applied to generate segmentation masks for the entire endothelial dataset. This image analysis workflow consisted of two primary steps: (1) detection of

epithelial remnants using the trained WEKA classifier, and (2) edge detection based on the nuclei channel.

For epithelial remnants detection, the trained WEKA model was utilized to produce a probability map indicating the likelihood of epithelial remnant presence. Regions with probability values exceeding 0.5 and an area threshold of at least 1000 μm² were classified as epithelial remnant regions[5] (**Supplementary Fig. S8A**). For edge detection via nuclei channel, a hard threshold of 2000 was applied to the MIP image of the nuclei channel to delineate edges. Morphological closing and opening operations were subsequently performed using an octagonal structuring element with a radius of 3. These operations aimed at removing small artifacts from the foreground and background (**Supplementary Fig. S8B**). The final mask was generated by subtracting the mask obtained in the second step (edge detection via nuclei channel) from the mask derived in the first step (epithelial remnants detection). This subtraction isolated the epithelial regions by removing the edge-associated regions, yielding a refined mask for subsequent analysis (**Supplementary Fig. S8C**).

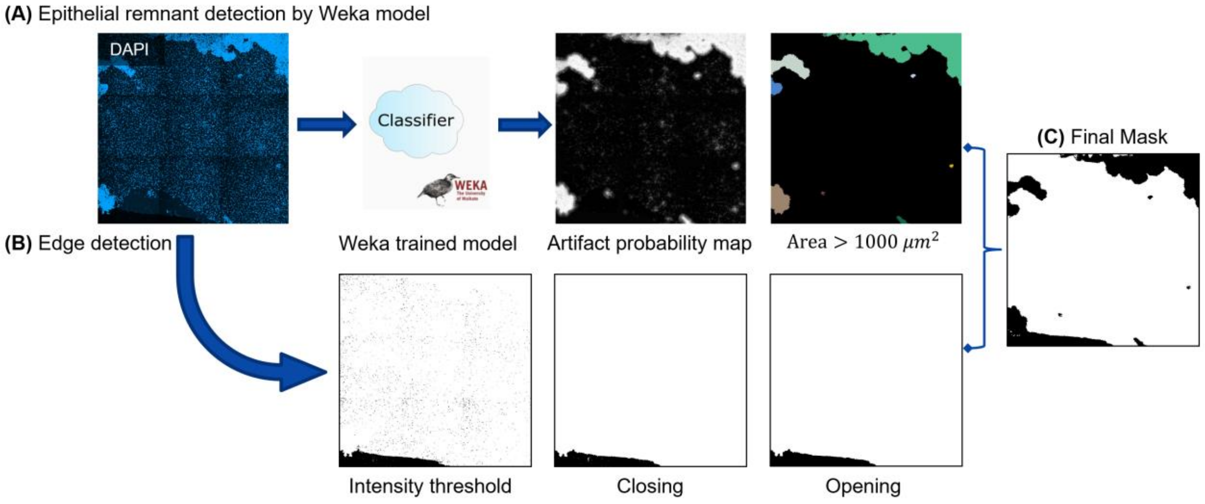

Supplementary Figure S8. Mask extraction workflow for endothelial image analysis. (A) Detection of epithelial remnants using the trained WEKA classifier. The classifier generates a probability map, with regions exhibiting a probability>0.5 and area>1000 μm² classified as epithelial remnant regions. **(B)** Edge detection based on DAPI intensity. A hard intensity threshold is applied, followed by morphological closing and opening operations to remove foreground and background artifacts, respectively. **(C)** Generation of the final mask by subtracting the binary mask obtained in (B) from the binary mask obtained in (A), thus isolating regions free of artifacts for further analysis.

Automated analysis of IoC image data in JIPipe

The primary analyses were fully developed in JIPipe[6]. Multi-channel z-stacks of epithelial and endothelial cell layers were imported, annotated, and separated into individual channels, including macrophages, ZO-1, nuclei, and BF. Three distinct analysis compartments were defined to simultaneously target specific features within the epithelial and endothelial datasets including the damage assessment of the tight junction protein ZO-1 in the epithelial cell layer, and the pixel-based evaluation of epithelial tissue thickness. In contrast, image analysis of the endothelial cell layer focused on quantifying the cell density and morphometrics of endothelial cells and macrophages. The analysis of the tissue thickness was initially implemented as ImageJ macros in one of our previous works[7]. In the current study, we have integrated these ImageJ macros into the JIPipe ImageJ macro node, maintaining the exact parameters used previously, while the remaining analyses utilize JIPipe nodes. Detailed descriptions of each analysis compartment are outlined below.

Loss of tight junction protein ZO-1 (epithelial analysis): First, maximum intensity projection (MIP) of the ZO-1 channel was generated to ensure that all slices in the z-stack, especially those from the crypt area, were accurately represented in the resulting 2D image (**Supplementary Fig. S9A**). A manual intensity threshold of 3000 was applied, whereby pixels with values ≤ 3000 were segmented as regions of tissue loss or damage (**Supplementary Fig. S9B**). Next, regions of interest (ROIs) smaller than 500 µm² were excluded to focus on sufficiently large areas of damage. this threshold was determined based on visual inspection. (**Supplementary Fig. S9C**). A closing operation using a square structural element of size 10 pixels was applied to the mask, removing dark spots within the damaged areas (**Supplementary Fig. S9D-E**). Finally, the processed mask was multiplied by the Weka-generated chip mask to exclude edge artifacts and refine the final damage mask (**Supplementary Fig. S98F**). The damage area fraction was calculated by normalizing the tissue damage area to the total chip area. In cases where two non-overlapping images were acquired from the same chip, the total damage area was normalized by the sum of the corresponding chip areas to ensure a comparable number of data points across conditions.

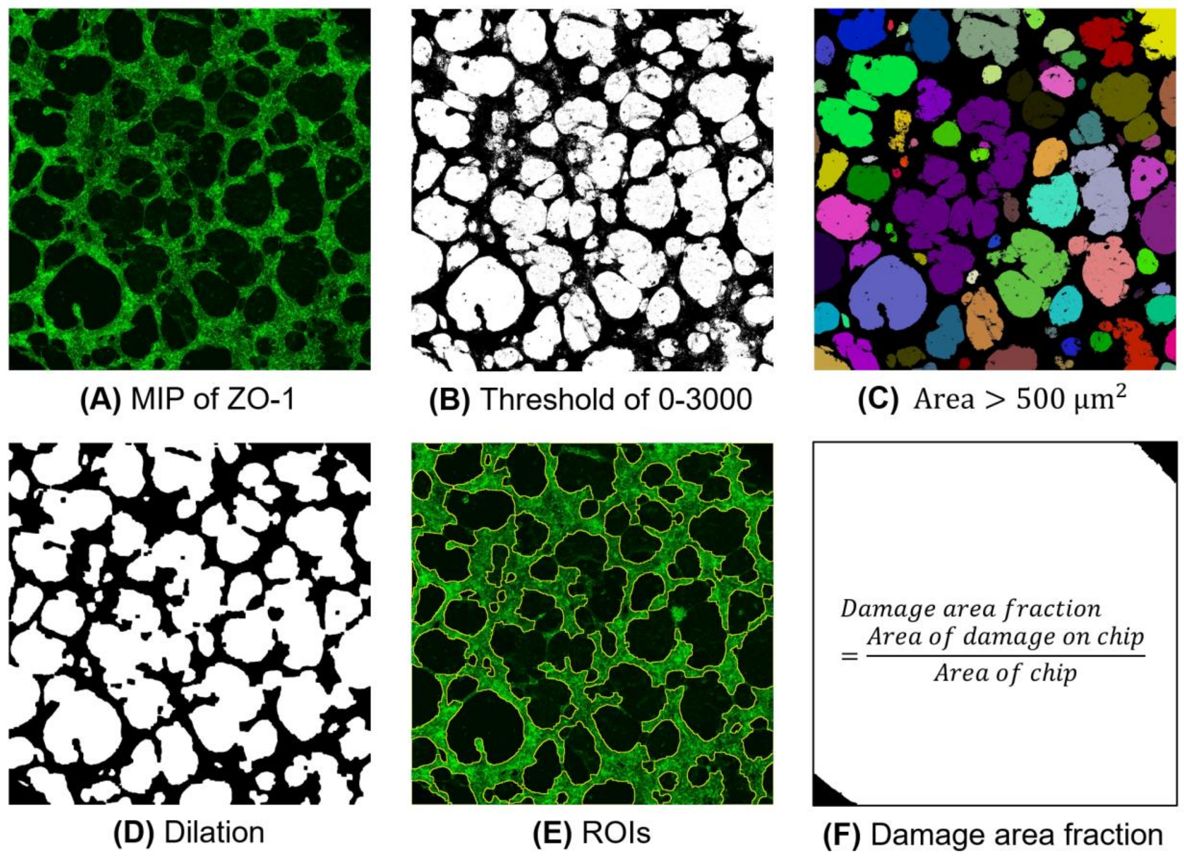

Supplementary Figure S9. ZO-1 damage area fraction. (A) Z-projection of the ZO-1 channel. **(B)** Binarization of damage areas through thresholding (0–3000 intensity range). **(C)** Damage area filtering, with regions larger than 500 μm² considered as significant for damage. **(D)** Dilation operation applied to fill dark spots within the damaged regions. **(E)** Damaged areas outlined in yellow. **(F)** Calculation of the damage area fraction by dividing the total ZO-1 damage area by the total chip area.

Tissue architecture (epithelial analysis): The 3D structure of the tissue was characterized by subtracting the interpolated epithelial tissue position from the membrane position at each x-y coordinate, resulting in a heatmap where each pixel value represents the epithelial tissue thickness at that specific location. Subsequently, tissue thickness measurements were obtained from 10,000 randomly selected positions within each image to provide a representative assessment. To validate the automated epithelial tissue thickness measurements, manual annotations were compared with automated results. Ten images were randomly selected, and three random 20×20 μm regions were defined within each image. Because single-point thickness estimation is not feasible by visual inspection, these regions represented the smallest areas consistently containing at least one nucleus. Tissue thickness

was manually measured within each region and compared to the mean automated thickness measured in the corresponding areas. The comparison showed good agreement between automated and manual measurements, with an R^2 of 0.82 and a mean absolute error (MAE) of 1.74 µm (**Supplementary Fig. S10-S11**).

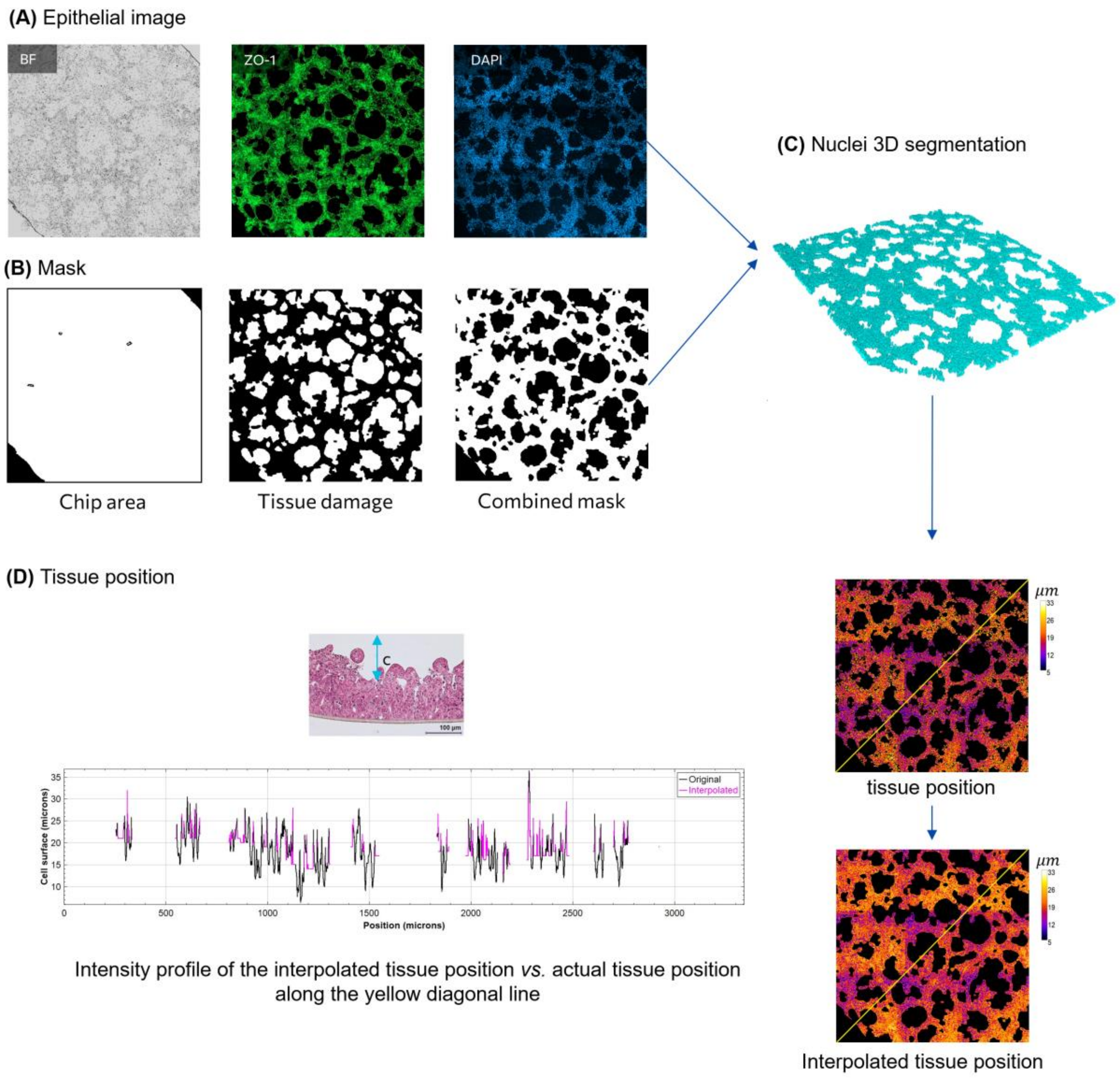

Supplementary Figure S10. Quantification of epithelial tissue position[7]. (A) 3D multi-channel images of the epithelial compartment including BF, ZO-1 and DAPI. **(B)** Mask of the chip area, excluding edges and artifacts, generated from the BF channel predicted by the trained Weka classifier. A separate mask for tissue damage was created based on signal loss in the ZO-1 channel. These masks were combined to generate a comprehensive mask for subsequent tissue architecture quantification. **(C)** 3D binarization of the nuclei channel, constrained by the combined mask to exclude artifacts and regions of tissue damage. **(D)** Tissue position map (indicated as cyan arrow on Caco-2 cell layers[8]) derived from the nuclei binarization and interpolated to bridge gaps between nuclei. The intensity profile along the

yellow line illustrates the interpolation of nuclei-to-nuclei distances while preventing unwanted interpolation in areas of tissue damage through mask integration.

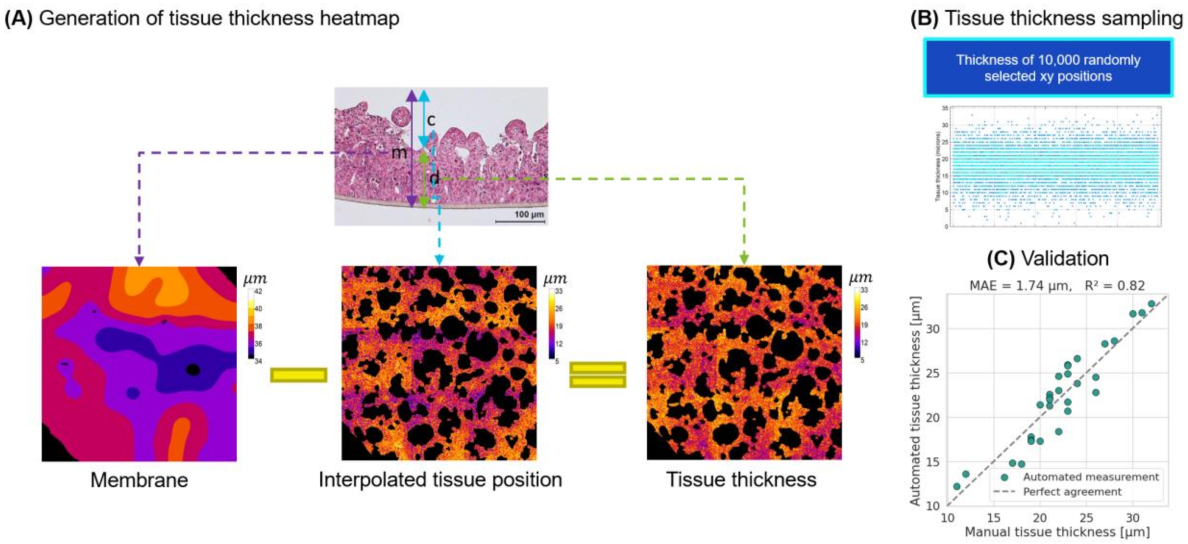

Supplementary Figure S11. Quantitative reconstruction of epithelial tissue. (A) The tissue structure map is obtained by subtracting the interpolated epithelial position from the membrane position, generating a heatmap of tissue thickness at all points. **(B)** Measurements from 10,000 randomly selected positions represent the sample. **(C)** Validation plot shows comparison between manually annotated and automated epithelial tissue thickness measurements. Each data point represents a 20×20 µm region sampled from 10 randomly selected images. The dashed line indicates perfect agreement.

Cells density and morphometrics (endothelial analysis): Due to imperfect tile stitching by the microscope, cells located at tile borders occasionally appeared duplicated or misaligned. To correct this, individual tiles of the nuclei and macrophage channels were manually cropped by defining precise XY coordinates based on visual inspection. The cropped tiles were then recombined using the *Combine* command in Fiji to produce correctly aligned images (**Supplementary Fig. S12A-B**). The corrected images were then saved and imported into JIPipe, along with the WEKA-predicted masks, for subsequent main analysis. First, an MIP was generated for both the nuclei and macrophage channels. The nuclei channel then underwent illumination correction using the *Illumination Correction 2D* node in JIPipe, which applied a Gaussian filter to estimate maximum pixel intensity, followed by normalization of all pixel values based on this maximum. This correction minimized uneven lighting across tiles, resulting in a more uniform image (**Supplementary Fig. S12C**). Illumination correction was not applied to the macrophage channel, as the macrophages exhibited a weak signal, thus applying the correction risked amplifying background noise.

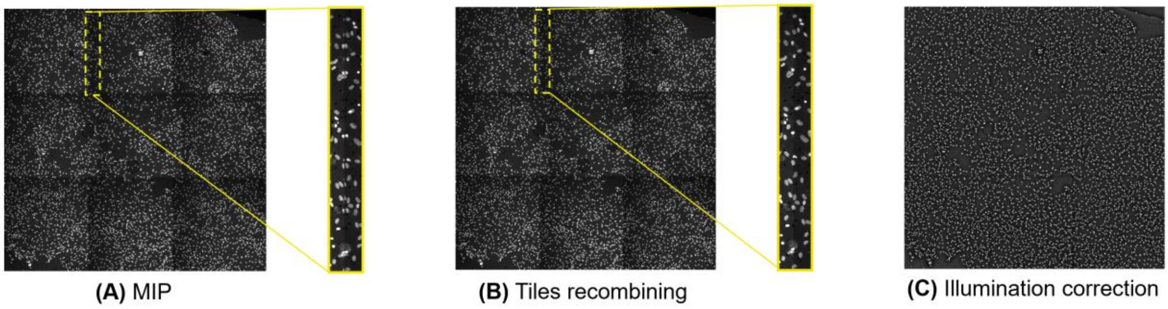

Supplementary Figure S12. Preprocessing of endothelial nuclei images. (A) MIP of the endothelial nuclei channel, illustrating misalignment of nuclei along tile borders. **(B)** Cropped and recombined tiles, using manually defined XY coordinates, which effectively corrects for microscope stitching artifacts. **(C)** Illumination correction applied to minimize uneven lighting across tiles, resulting in a more uniform image.

To segment the nuclei, a pretrained Cellpose 2.0 model[9] within JIPipe was applied using the *Cytoplasm* model with an average object diameter of 15 pixels. Because the model was previously trained on a large and diverse dataset of more than 70,000 manually annotated cellular regions, it enables robust and accurate segmentation without requiring additional training on the present dataset. To ensure that the network did not incorrectly identify background features, such as membrane pores, as nuclei, an intensity threshold of 5000 was applied, retaining only ROIs with average intensity values above this threshold. Following this, detected nuclei objects that overlapped by more than 90% with the predefined mask were retained, while those with less overlap were discarded, as they were likely to represent DAPI signals from epithelial remnants rather than actual endothelial nuclei (**Supplementary Fig. S13**).

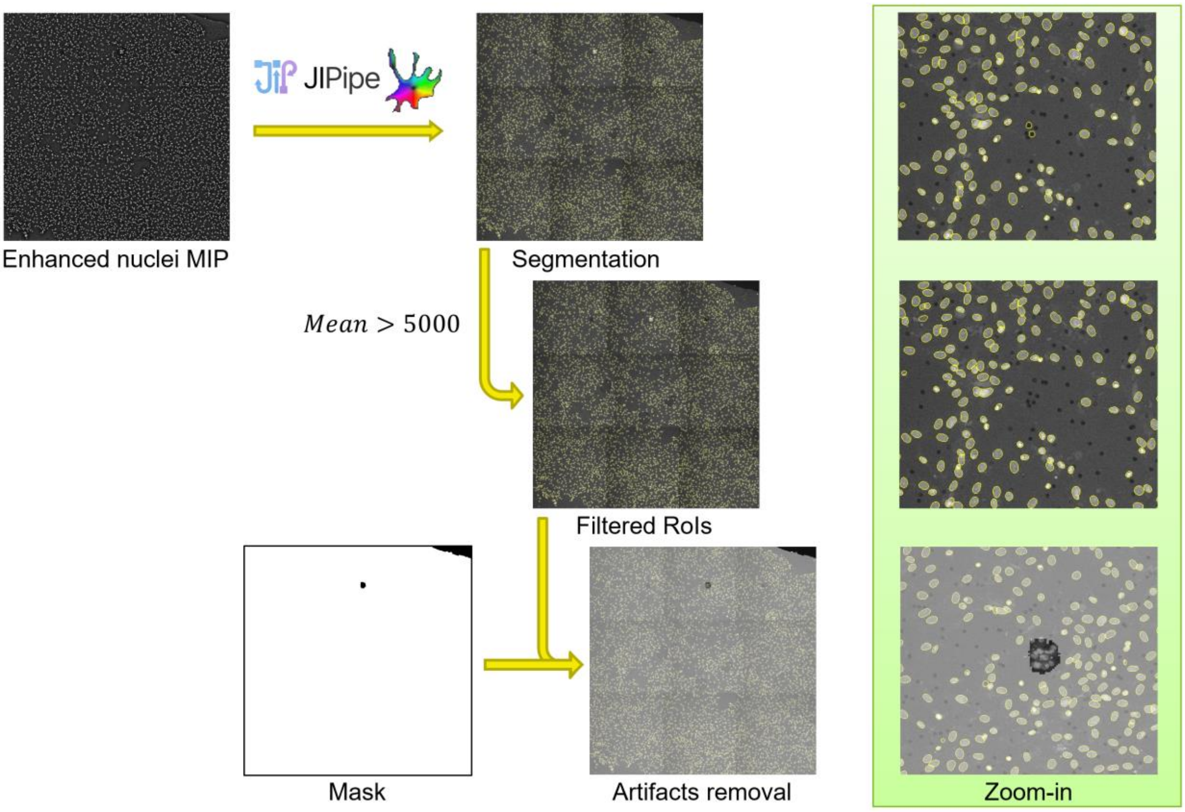

Supplementary Figure S13. Nuclei detection by Cellpose in JIPipe. A pretrained Cellpose model within JIPipe was applied for nuclei segmentation. To prevent misidentification of background features, such as membrane pores, an intensity threshold of 5000 was applied. Filtered ROIs with more than 90% overlap with the endothelial mask were retained, while those with less overlap were discarded to avoid including DAPI signals from epithelial remnants. The segmentation workflow is illustrated, with zoomed-in images at each step.

The workflow for macrophage segmentation followed the same approach, with the exception that the *Cytoplasm2* model was used in the Cellpose configuration, with an average object diameter of 20 pixels (**Supplementary Fig. S14**). Finally, using the *Extract ROI Statistics* node in JIPipe, morphological characteristics were extracted for each segmented channel. The total number of nuclei and macrophages was calculated, and cell density for each image was determined by normalizing the total number of ROIs to the area of the chip. Additionally, the mean fluorescence intensity (MFI) of macrophages was determined as the sum of fluorescence intensity over macrophage segmentations, normalized by the total number of macrophage pixels.

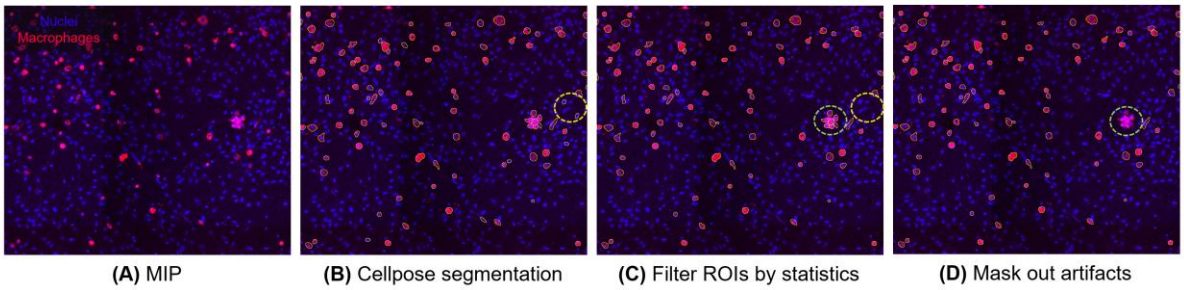

Supplementary Figure S14. Macrophage detection by Cellpose. (A) MIP of the macrophage channel overlaid with the nuclei channel. **(B)** Macrophage segmentation performed using a pretrained Cellpose model within JIPipe. **(C)** Filtered ROIs with intensity values greater than 5000 to avoid background identification (indicated by the yellow dashed circle). **(D)** Masking of artifacts from epithelial remnants (indicated by the green dashed circle).

Statistical analysis

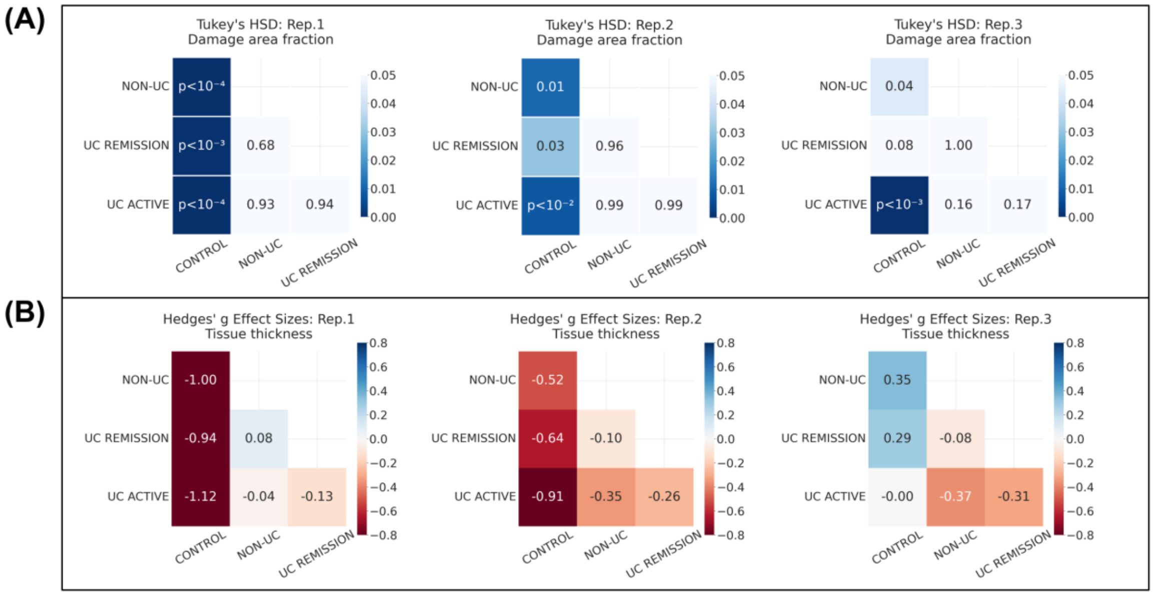

Supplementary Figure S15. Statistical significance and effect size analysis of epithelial tissue damage and thickness across replicates. **(A)** Tukey's HSD test on damage area fraction; p-value < 0.05 are considered statistically significant. **(B)** Hedges' *g* effect sizes for tissue thickness. Given the large sample size, effect size was used instead of p-values to provide a more reliable measure of magnitude of differences between groups. A value of approximately g = 0.2 indicates a small effect, g = 0.5 a medium effect, and g ≥ 0.8 a large effect.

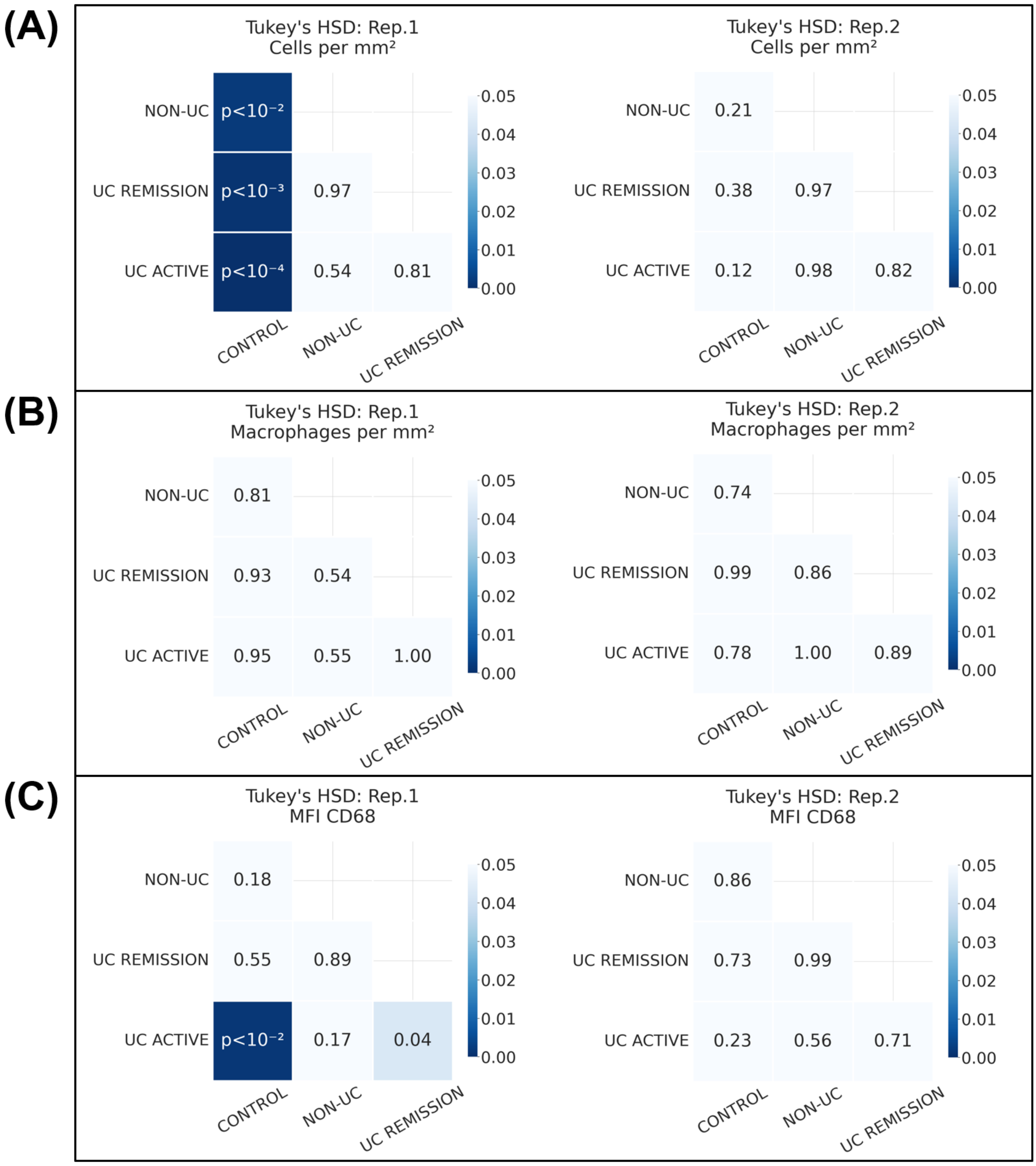

Supplementary Figure S16. Statistical significance in endothelial and macrophage cell densities and fluorescence characteristics across replicates. Tukey's HSD test on **(A)** endothelial cell density, **(B)** macrophage cell density and **(C)** macrophage MFI. p-value < 0.05 are considered statistically significant.

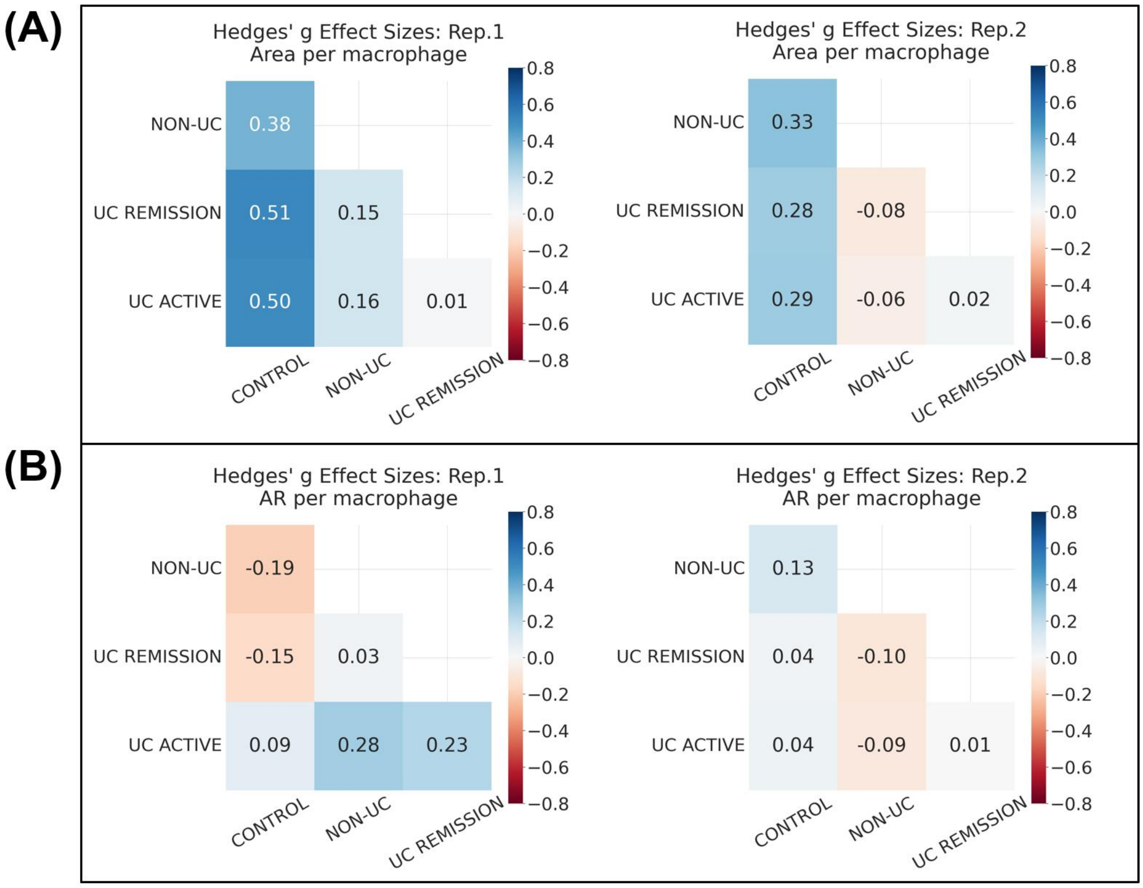

Supplementary Figure S17. Effect size analysis of macrophage area and aspect ratio across replicates. Hedges' g effect sizes are shown for **(A)** macrophage area and **(B)** aspect ratio (AR). A value of around g = 0.2 is considered to be a low effect, g = 0.5 suggests a medium effect, and g = 0.8 or larger signifies a large effect.

Tables

Condition/patient group	Total	UC remission	UC active disease	Non-IBD
	N			
All patients	10	4	6	5
Sex<br>*Female*<br>*Male*	10<br>6<br>9	 2<br>2	 2<br>4	 2<br>3
Age (years)*		33.5 (31; 36.25)	35.5 (33.25; 46.75)	26 (23; 37)
Age at diagnosis (years)*		27 (25.5; 29)	32.5 (29; 39)	-
BMI (kg/m^2)*		22 (21; 25.25)	25.5 (23.5; 26)	23 (22; 26)
CRP [mg/L]*		1.6 (0.9; 5.85)	12.95 (6.45; 16.375)	-
Leukozyten [Gpt/L]*		8.55 (7.55; 8.7)	11.25 (7.5; 12)	-
Calprotectin [µg/g]*		52.7 (23.325; 123.775)	2000 (2000; 2641.1)	-
Mayo-Score (MS)*<br>*Partial MS*<br>*Endoskopic MS*		 0 (0; 0.75)<br>0 (0; 0)	 7 (5.5; 7)<br>3 (3; 3)	-
Extent (Montreal)<br>*E1*<br>*E2*<br>*E3*	10<br>0<br>4<br>6	 0<br>2<br>2	 0<br>2<br>4	-

Supplementary Table S1. Characteristics of the study population. Clinical parameters of all study participants and stratified patient groups including ulcerative colitis (UC) patients in remission, UC patients with active disease, and non-IBD controls. Continuous variables* are presented as median with interquartile range (Q1–Q3). The extent of UC was classified according to the Montreal classification (E1–E3), referring to the macroscopic extent of intestinal inflammation. Abbreviations: BMI, body mass index; CRP, C-reactive protein; MS, Mayo Score.

Solution and cell media	Composition
EC-medium with all supplements	1. Endothelial Cell Growth Medium (PromoCell, Heidelberg, Germany)<br>2. Supplement mix (PromoCell)<br>3. 10% autologous human serum<br>4. 10 ng/mL M-CSF (PeproTech)<br>5. 10 ng/mL GM-CSF (PeproTech)<br>6. 100 U/mL penicillin and 100 µg/mL streptomycin (Pen/Strep) (Gibco, Darmstadt, Germany)
GC-medium	1. DMEM high glucose (4.5 g/L) (Gibco, Darmstadt, Germany)<br>2. 10% foetal calf serum (Gibco)<br>3. 1% GlutaMax (Gibco)<br>4. 1% MEM non-essential amino acids (Gibco)<br>5. 10 µg/mL human holo-transferrin (Sigma Aldrich, Darmstadt, Germany)<br>6. 1 mM sodium pyruvate (Gibco)<br>7. 20 µg/mL gentamicin (Gibco)
Monocyte differentiation medium	▪ X-VIVO 15 Serum-free Hematopoietic Cell Medium (Lonza, Basel, Switzerland)<br>▪ 10% autologous human serum<br>▪ 10 ng/mL M-CSF<br>▪ 10 ng/mL GM-CSF<br>▪ 100 U/mL penicillin and 100 µg/mL streptomycin
Trypsin/EDTA solution (detachement reagent)	In PBS -/- (Gibco)<br>▪ 0.25% trypsin (Gibco)<br>▪ 1 mM EDTA (Invitrogen)
Collagen solution	In PBS +/+ (Gibco)<br>▪ 0.5 mg/mL collagen IV (Sigma Aldrich)<br>▪ 0.5 mM acetic acid (Sigma Aldrich)
Lidocaine/EDTA solution	In PBS -/-<br>▪ 4 mg/mL lidocaine (Sigma Aldrich)<br>▪ 5 mM EDTA (Invitrogen)
FITC-Dextran/media solution	1 mg/mL FITC-Dextran (Sigma Aldrich) in GC-medium with phenol red free DMEM (Gibco)
Blocking and permeabilizing solution	In PBS +/+<br>▪ 3% normal donkey serum (NDS) (BIOZOL, Hamburg, Germany)<br>▪ 0.1% saponin (Sigma Aldrich)
Wash solution	0.1% saponin in PBS +/+
IF-staining solution	In PBS +/+<br>▪ 0.3% NDS<br>▪ 0.1% saponin<br>▪ primary or secondary antibodies or DAPI
Isolation-buffer	In PBS -/-<br>▪ 1 mg/mL bovine serum albumin fraction V (Sigma Aldrich)<br>▪ 2 mM EDTA

Supplementary Table S2. Solutions and cell media used for the experiments and their composition.

		Dilution	Manufacturer and location
**Primary antibody**	Anti-VE-Cadherin<br>(Goat anti-Human)	1:100	R&D Systems Inc., Minneapolis, US
	Anti-CD68<br>(Rabbit anti-Human)	1:100	Cell Signaling Technology Inc., Danvers, US
	Anti-E-Cadherin<br>(Rat anti-Human)	1:100	Sigma-Aldrich, Darmstadt, Germany
	Anti-ZO1<br>(Rabbit anti-Human)	1:100	Invitrogen (Thermo Fisher Scientific Inc.), Waltham, USA
	Anti-CD103<br>(Mouse Anti-Human)	1:100	BD Biosciences, Franklin Lakes, US
**Secondary antibody**	Alexa Fluor® 488<br>(Donkey Anti-Rabbit)	1:200	Jackson ImmunoResearch Europe Ltd., Ely, UK
	Alexa Fluor™ 647<br>(Donkey anti-Rabbit)	1:200	Invitrogen (Thermo Fisher Scientific Inc.), Waltham, US
	Alexa Fluor™ 647<br>(Donkey anti-Mouse)	1:200	Invitrogen (Thermo Fisher Scientific Inc.), Waltham, US
	Cy™3<br>(Donkey Anti-Goat)	1:100	Jackson ImmunoResearch Europe Ltd., Ely, UK
	Cy™3<br>(Donkey Anti-Rat)	1:200	Jackson ImmunoResearch Europe Ltd., Ely, UK
**Nuclear counterstain**	DAPI (4′,6-diamidino-2-phenylindole)	1:2.000	Invitrogen (Thermo Fisher Scientific Inc.), Waltham, US

Supplementary Table S3. IF-staining panel with all antibodies and DAPI. The indicated dilutions refer to the IF-staining solution from Table 1.

Cyto-/chemokine	Time [hours]	nU vs. Ua	Ur vs. Ua	nU vs. Ur	C vs. nU	C vs. Ur	C vs. Ua
**Interleukin-1β**	t=24	0.0095	0.019	0.6857	0.1905	0.0635	0.0043
	t=48	0.0095	0.019	>0.9999	0.381	0.1667	0.0043
	t=72	0.0095	0.019	0.8857	0.556	0.1667	0.0043
**Interleukin-6**	t=24	0.0381	0.1714	0.3429	0.0317	0.0159	0.0043
	t=48	0.019	0.1714	0.2	0.0159	0.0159	0.0043
	t=72	0.0095	0.0095	>0.9999	0.0159	0.0159	0.0043
**Interleukin-8**	t=24	0.0667	0.4762	0.2	0.0159	0.0159	0.0043
	t=48	0.0381	>0.9999	0.1143	0.0159	0.0159	0.0043
	t=72	0.0381	0.0095	0.8857	0.0159	0.0159	0.0043
**MCP-1**	t=24	0.0095	0.019	0.3429	0.0159	0.0159	0.0043
	t=48	0.019	0.4762	0.1143	0.0159	0.0159	0.0043
	t=72	0.019	0.0667	0.6857	0.0159	0.0159	0.0043
**Interferon-α2**	t=24	0.019	0.019	0.3429	0.4127	0.0635	0.0043
	t=48	0.019	0.019	0.7714	0.2857	0.0635	0.0043
	t=72	0.0381	0.0381	0.8857	0.1111	0.0317	0.0043
**Interferon-γ**	t=24	0.019	0.0381	0.3429	0.5159	0.0635	0.0043
	t=48	0.019	0.0095	>0.9999	0.619	0.0952	0.0043
	t=72	0.0381	0.019	0.9714	0.2143	0.0873	0.0043
**TNF-α**	t=24	0.0667	0.7619	0.1143	0.7857	0.0159	0.0043
	t=48	0.1143	0.1714	0.9429	0.3889	0.0159	0.0043
	t=72	0.2571	0.019	0.8857	0.0566	0.0079	0.0043
**Interleukin-10**	t=24	0.1143	0.2571	0.4857	0.4524	0.0159	0.0043
	t=48	0.1714	0.3524	0.7714	0.1111	0.0556	0.0152
	t=72	0.4762	0.2571	0.6857	0.0952	0.1349	0.0043
**Interleukin-12 (p70)**	t=24	0.6095	0.7619	0.3429	0.4127	0.317	0.0087
	t=48	0.7619	0.9143	>0.9999	0.2857	0.1111	0.0173
	t=72	0.8048	0.9143	0.8857	0.0476	0.0159	0.0087
**Interleukin-17A**	t=24	0.4762	0.9143	0.4857	0.1111	0.0476	0.0087
	t=48	0.0381	0.5143	0.4857	0.1746	0.1111	0.0033
	t=72	0.9714	0.9143	0.6857	0.0476	0.0079	0.0087
**Interleukin-18**	t=24	0.3524	0.9143	0.4857	0.556	0.1111	0.0173
	t=48	0.4762	0.4762	0.8857	0.556	0.2857	0.0823
	t=72	0.7619	0.9143	0.6857	0.1905	0.1111	0.0043
**Interleukin-23**	t=24	0.0667	0.3524	0.3429	0.1905	0.0159	0.0043
	t=48	0.019	0.0667	0.3429	0.0156	0.0317	0.0043
	t=72	0.0429	0.0381	0.6857	0.0159	0.0159	0.0043
**Interleukin-33**	t=24	0.1714	0.1143	0.4857	0.9048	0.1111	0.0043
	t=48	0.0501	0.5142	0.3429	0.1905	0.1667	0.0152
	t=72	0.1143	0.3524	0.3429	0.0635	0.0159	0.0043

Supplementary Table S4. Statistical significance of cytokine and chemokine measurements in the epithelial compartment of the intestine-on-chip. Calculations were performed using the non-parametric Kruskal–Wallis test with Dunn's multiple comparisons test. Abbreviations: C = control, nU = non-UC, Ur = UC remission, Ua = UC active. Data from one representative experiment from three independent immunological replicates are shown.

Cyto-/chemokine	Time [hours]	nU vs. Ua	Ur vs. Ua	nU vs. Ur	C vs. nU	C vs. Ur	C vs. Ua
**Interleukin-1β**	t=24	0.0095	0.0095	0.9714	0.3333	0.5635	0.0043
	t=48	0.0095	0.0095	0.2286	0.1143	0.2063	0.0043
	t=72	0.0095	0.0095	0.5429	0.9841	0.5635	0.0043
**Interleukin-6**	t=24	0.0667	0.3524	0.6857	0.0159	0.0159	0.0043
	t=48	0.0381	0.6095	0.1143	0.0317	0.0159	0.0043
	t=72	0.0095	0.0095	>0.9999	0.0159	0.0159	0.0043
**Interleukin-8**	t=24	0.0159	0.4524	0.6857	0.0159	0.0159	0.0079
	t=48	0.0159	0.0763	0.2	0.0317	0.0159	0.0079
	t=72	0.0159	0.0635	0.3429	0.0159	0.0159	0.0079
**MCP-1**	t=24	0.0667	0.7619	0.02	0.1905	0.0317	0.0087
	t=48	0.019	0.8857	0.1143	0.0317	0.0159	0.0043
	t=72	0.0095	0.0667	0.1143	0.0159	0.0159	0.0043
**Interferon-α2**	t=24	0.6095	>0.9999	0.8857	0.254	0.0556	0.0173
	t=48	0.2571	0.6095	0.2	0.3175	0.0317	0.0087
	t=72	0.1143	0.6095	0.6857	0.0476	0.0476	0.0043
**Interferon-γ**	t=24	0.1952	0.6476	0.0571	0.5714	0.2857	0.0952
	t=48	0.281	0.9143	0.0857	0.5714	0.0476	0.0693
	t=72	0.381	0.9619	0.6857	>0.9999	0.373	0.2251
**TNF-α**	t=24	0.4762	0.7619	0.7714	0.1905	0.3413	0.4633
	t=48	0.6571	0.9143	0.9909	0.2716	0.0159	0.0143
	t=72	0.6571	0.5143	0.6286	0.4206	0.7063	0.2143
**Interleukin-10**	t=24	0.8	0.3333	0.3714	0.1032	0.9048	0.1667
	t=48	0.019	0.1143	0.7429	0.8254	0.9999	0.3939
	t=72	0.6381	0.6381	>0.9999	>0.9999	>0.9999	0.1333
**Interleukin-12 (p70)**	t=24	0.5143	0.381	0.3429	0.1429	0.3413	0.3225
	t=48	0.5095	0.1571	0.3429	0.0159	0.0476	0.3095
	t=72	0.4381	0.5714	0.9714	0.5714	0.5079	>0.9999
**Interleukin-17A**	t=24	0.0286	0.3095	0.5429	0.8095	0.5238	0.0455
	t=48	0.0238	0.7619	0.3714	0.1716	0.7619	0.3889
	t=72	0.0286	0.0286	>0.9999	0.9268	>0.9999	0.0455
**Interleukin-18**	t=24	0.1714	0.6095	0.8857	0.0476	0.0079	0.0043
	t=48	0.0714	0.9143	0.2	0.1032	0.0238	0.0152
	t=72	0.2571	0.3524	0.6857	0.0476	0.0079	0.0043
**Interleukin-23**	t=24	0.2571	0.7619	0.8857	0.22619	0.9524	0.0433
	t=48	0.4762	0.9143	0.1429	0.5719	0.5556	0.1028
	t=72	0.2571	0.3524	0.8857	0.5952	0.3968	0.026
**Interleukin-33**	t=24	0.381	0.5833	>0.9999	0.2	0.2	0.0333
	t=48	0.1071	0.9048	0.2	0.4857	0.0571	0.0381
	t=72	0.6667	0.3571	>0.9999	0.1714	0.1714	0.0048

Supplementary Table S5. Statistical significance of cytokine and chemokine measurements in the endothelial compartment of the intestine-on-chip. Calculations were performed using the non-parametric Kruskal–Wallis test with Dunn's multiple comparisons test. Abbreviations: C = control, nU = non-UC, Ur = UC remission, Ua = UC active. Data from one representative experiment from three independent immunological replicates are shown.

References

1. Elmentaite R, Kumasaka N, Roberts K, Fleming A, Dann E, King HW, et al. Cells of the human intestinal tract mapped across space and time. Nature. 2021;597(7875):250–5. doi:10.1038/s41586-021-03852-1

2. Arganda-Carreras I, Kaynig V, Rueden C, Eliceiri KW, Schindelin J, Cardona A. Trainable Weka Segmentation: a machine learning tool for microscopy pixel classification. Bioinformatics. 2017;33(15):2424–6. doi:10.1093/bioinformatics/btx180

3. Schindelin J, Arganda-Carreras I, Frise E, Kaynig V, Longair M, Pietzsch T. Fiji: an open-source platform for biological-image analysis. Nat Methods. 2012;9:676–82. doi:10.1038/nmeth.2019

4. Legland D, Arganda-Carreras I, Andrey P. MorphoLibJ: integrated library and plugins for mathematical morphology with ImageJ. Bioinformatics. 2016;32:3532–4. doi:10.1093/bioinformatics/btw413

5. Domander R, Felder AA, Doube M. BoneJ2 - refactoring established research software. Wellcome Open Res. 2021;6:37. doi:10.12688/wellcomeopenres.16619.2

6. Gerst R, Cseresnyes Z, Figge MT. JIPipe: visual batch processing for ImageJ. Nat Methods. 2023;20:168–9. doi:10.1038/s41592-022-01744-4

7. Kaden T, Alonso-Roman R, Akbarimoghaddam P, Mosig AS, Graf K, Raasch M. Modeling of intravenous caspofungin administration using an intestine-on-chip reveals altered Candida albicans microcolonies and pathogenicity. Biomaterials. 2024;307:122525. doi:10.1016/j.biomaterials.2024.122525

8. Maurer M, Gresnigt MS, Last A, Wollny T, Berlinghof F, Pospich R. A three-dimensional immunocompetent intestine-on-chip model as in vitro platform for functional and microbial interaction studies. Biomaterials. 2019;220:119396. doi:10.1016/j.biomaterials.2019.119396

9. Stringer C, Wang T, Michaelos M, Pachitariu M. Cellpose: a generalist algorithm for cellular segmentation. Nat Methods. 2021;18:100–6. doi:10.1038/s41592-020-01018-x
